# Molecular profiling of the intestinal mucosa and immune cells of the colon by multi-parametric histological techniques

**DOI:** 10.1038/s41598-021-90761-y

**Published:** 2021-05-28

**Authors:** Łukasz Zadka, Karolina Chrabaszcz, Igor Buzalewicz, Ewelina Wiercigroch, Natalia Glatzel-Plucińska, Łukasz Szleszkowski, Agnieszka Gomułkiewicz, Aleksandra Piotrowska, Krzysztof Kurnol, Piotr Dzięgiel, Tomasz Jurek, Kamilla Malek

**Affiliations:** 1 Histology and Embryology Division, Department of Human Morphology and Embryology, Wroclaw Medical University, Chałubińskiego 6a, 50-368 Wrocław, Poland; 2 Faculty of Chemistry, Jagiellonian University in Krakow, Krakow, Poland; 3 Bio-Optics Group, Department of Biomedical Engineering, Faculty of Fundamental Problems of Technology, Wrocław University of Science and Technology, 27 Wybrzeże S. Wyspiańskiego St., 50-370, Wroclaw, Poland; 4 Department of Forensic Medicine, Forensic Medicine Unit, Wroclaw Medical University, Wroclaw, Poland; 5 Department of General and Oncological Surgery, Wroclaw Medical University, Wrocław, Poland

**Keywords:** Gastroenterology, Gastrointestinal system, Large intestine, Colon, Molecular medicine, Immunopathogenesis, Lymphoid tissues, Mucosal immunology, Chemical biology

## Abstract

The impact of the post-mortem interval (PMI) on the optical molecular characteristics of the colonic mucosa and the gut-associated lymphoid tissue (GALT) were examined by multi-parametric measurements techniques. Inflammatory cells were identified by immunohistochemical staining. Molecular parameters were estimated using the Raman spectroscopy (RS) and Fourier Transform Infrared (FTIR) spectroscopic imaging. The 3D refractive index (3D-RI) distributions of samples were determined using the digital holographic tomography. The distribution of immune cells between post-mortem (PM) and normal controls did show significant differences for CD4 (*P* = 0.0016) or CD8 (*P* < 0.0001), whose expression level was decreased in PM cases. No association was found between individual PMI values and inflammatory cell distribution. However, there was a tendency for a negative correlation between CD4^+^ cells and PMI (r = − 0.542, *P* = 0.032). The alterations ongoing in post-mortem tissue may suggest that PMI has a suppressive effect on the effector properties of the cell-mediated immunity. Moreover, it was confirmed that spectroscopic and digital holotomographic histology are also a useful technique for characterization of the differences in inflammation of varying intensity and in GALT imaging in a solid tissue. Anatomical location of immune cells and methods of tissue fixation determine the molecular and optical parameters of the examined cases.

## Introduction

Pathological researches based on post-mortem samples provide accessibility to all types of human tissues, which offer better understanding of the disease^1^. Currently, a research autopsy is commonly used, since it allows to expand the knowledge about the complex biology of cancer and the mechanism of metastasis formation^1^, thus there is a wide acceptance of donors’ relatives for the use of post-mortem samples for medical research^2^. These tissues are also extensively used in both clinical and forensic analyses, particularly in the case of toxicological tests^3,4^.

In turn, the constantly growing popularity of using human tissue from forensic autopsies in pathology is related to the fundamental question on the actual value of the obtained results. Not much is known about the molecular changes that occur in tissue under post-mortem conditions. In immunohistochemistry reactions (IHC), which are commonly used in routine clinical practice, even a delayed fixation of the collected sample may have a negative effect on the level of expression of tested biomarkers and thus can lead to misdiagnosis^5^. It is assumed that changes in molecular parameters may occur during the post-mortem interval (PMI) especially with regard to gene and protein expression level, which can lead to changes at the cellular response^1^.

As a component of the intestinal wall, the mucosa-associated lymphoid tissue (MALT) is a subtype of tissue with defense properties capable of inducing an immune response against specific antigens, which while present in the lumen of the ducts, maintain contact with the mucous membranes. Its morphology and properties depend on various features associated with organ specifications, anatomical location, patient age, environmental factors, lifestyle as well as with molecular features, which include, e.g., epigenetic differences^6^. In terms of the colonic mucosa, lymphoid aggregates of MALT demonstrate the functional and structural specificity and divide the mucosal layer into two main regions, i.e., the gut-associated lymphoid tissue (GALT) and GALT-free mucosal area. The clusters of immune cells (mostly B and T lymphocytes) that form lymphocytic follicles are the main form of GALT in the large intestine. Immune cells which infiltrate the intestinal mucosa are another form of GALT. The GALT-free mucosal area is formed by epithelial cells with secretory and absorptive functions^7^. GALT is also an important organ in pathology, as it is the starting point for some gastrointestinal tumors with specific tissue characteristics^7^.

The intestinal mucosa remains an important link between gut microbiota and the lymphoid tissue that in the case of disturbed homeostasis between these microenvironments can be the origin of cancer development^8^. Long-term inflammation of the colonic mucosa leads to molecular and structural changes, which are the key factors for developing colorectal cancer^9^. Furthermore, the routine histopathological diagnosis increasingly emphasizes the prognostic value of immune cell infiltrations^10^. The evaluation of immune cell immunophenotype in the solid tissue is also used in the immunological classification of tumors. However, these studies are based on the sole consideration of IHC staining^11^.

The importance of inflammatory cells in the pathogenesis of gastrointestinal disorders and technical difficulties encountered in the diagnosis of the inflamed tissue by a digital method should encourage the search for novel methodological solutions^12^. A spatial diversity of solid tissue and its heterogeneity are some of the key elements that limit the results in the process of discovering new biomarkers of diagnostic and prognostic significance. A complete attempt to identify new biomolecules which are present collectively in the tested sample by molecular analysis often makes it impossible to show their exact location and tissue distribution^13^.

In digital pathology, state-of-the-art techniques that could support the methods of routine staining are increasingly applied. Particular attention should be paid to some spectroscopic imaging techniques based on the detection of molecular vibrations, i.e. Fourier Transform Infrared (FTIR) and Raman (RS) spectroscopies which are non-destructive to biological samples, label-free and provide a holistic characterization of biomolecules present in the sample which undergo alterations. Both techniques are sensitive to biochemical composition of cells and tissues and their spectra indicate distribution of any molecular changes that occur due to inflammation, carcinogenesis or any pathological process^14–16^. They indeed indicate what molecular components contribute to distinguished tissue structures and what molecular changes occur in pathological processes. The obtained hyperspectral data from FTIR and Raman imaging, often thousands of spectra, are coded in false-color images which enable assessment of histologically recognized tissue structures and pathological changes based on their vibrational specific signatures. In addition, the interpretation of FTIR and Raman spectra indicates what groups of biomolecules contribute to this classification and in consequence what histological staining should be used to specify markers responsible for tissue transformation^17,18^. Since it is difficult to select proper immune/histochemical labels to identify compositional changes in tissues, FTIR and RS imaging techniques are powerful tools to support the target analysis of the complex biomatrix.

Recently, optical diffraction tomography or digital holotomography (DHT) has enabled the label-free quantitative three-dimensional (3D) imaging of different biological samples^19–25^. The DHT based on the limited-angle holographic tomography (LAHT)^22,26,27^ allows the reconstruction of the 3D refractive index (RI) distribution of a sample from multiple 2D digital holograms registered for various incident angles of the illuminating beam. Being an optical property of materials, the RI enables the characterization of the morphological properties, density changes or physiological and biochemical processes of living cells, tissues or microorganisms. Moreover, contrary to the fluorescence confocal microscopy, the DHT enables non-destructive, label-free 3D quantitative phase imaging^28^, lower phototoxicity and no photobleaching.

The aim of this study was to assess the molecular differences of normal colonic mucosa and lymphoid tissue in PMI. Post-mortem tissue fragments were compared with normal surgical margins obtained intraoperatively from the large intestine. IHC was used to evaluate the immunophenotype of the colonic mucosa. Spectroscopic methods such as FTIR and RS were employed in turn to evaluate the molecular differences of the examined tissues. These methods were also implemented as a new state-of-the-art technique to detect the immune cells and gut-associated lymphoid tissue. To the best of our knowledge, our study is the first to perform such analyses. Moreover, this study aimed to demonstrate the potential use of DHT in the histopathological examination of colorectal tissues. It was used to characterize the differences between the tissue samples taken from living patients and cadavers as well as to examine the colonic mucosa and lymphoid tissue in the post-mortem interval utilizing the optical property changes and to compare them in the context of the alternative methods commonly used for the histopathological examination or digital pathology. The obtained results and statistical analysis indicate that the DHT has perspectives to be used in digital pathology as a non-destructive and label-free 3D quantitative imaging technique. The results suggest that DHT exhibits high biological specificity in immune cell differentiation. The combination of different imaging and measurement techniques (DHT, RS, FTIR) used in this study allows the complex characterization of tissue samples based on their morphological and optical properties, as well as their chemical composition.

## Materials and methods

### Post-mortem samples of the intestinal mucosa

The intestinal tissue material was collected during forensic autopsies in the Department of Forensic Medicine in Wroclaw, Poland, in 2017. The inclusion criteria were sudden death, most often resulting from a traffic accident and relatively early post-mortem interval (PMI). The exclusion criteria were as follows: cancer, past medical history of inflammatory bowel disease, intestinal hemorrhage, intoxication and advanced decomposition of the body. Toxicological and histological examinations were performed prior to the inclusion in the study. Only patients who did not show any pathological signs suggestive of active disease status and/or any of the above exclusion criteria in the macro- and microscopic evaluation (assessment of the standardized preparations stained with the hematoxylin and eosin [H&E], laboratory and toxicological test results) were enrolled in the study. Tissue sections of 0.5–1 cm thickness were collected from each anatomical area. They were taken from the sigmoid and ascending colon, around 10 cm proximal to the right colic flexure and 5 cm distal from the sigmoid flexure. A total of sixteen tissue fragments were collected from eight patients who were enrolled in the study. All post-mortem samples were placed in biopsy cassettes and immediately immersed in paraffin. In addition, one colon tissue fragment around the sigmoid colon area was taken from each patient and then, immediately after collection, was placed in tubes and transported on ice at 4 °C in order to secure each sample at the deep-freezing temperature (T =  − 80 °C) for further molecular analysis. Due to the short distance between both departments the time of transport was no longer than 5 min. Each sample was collected in the summer.

### Normal colonic mucosa samples

**Figure 1:**
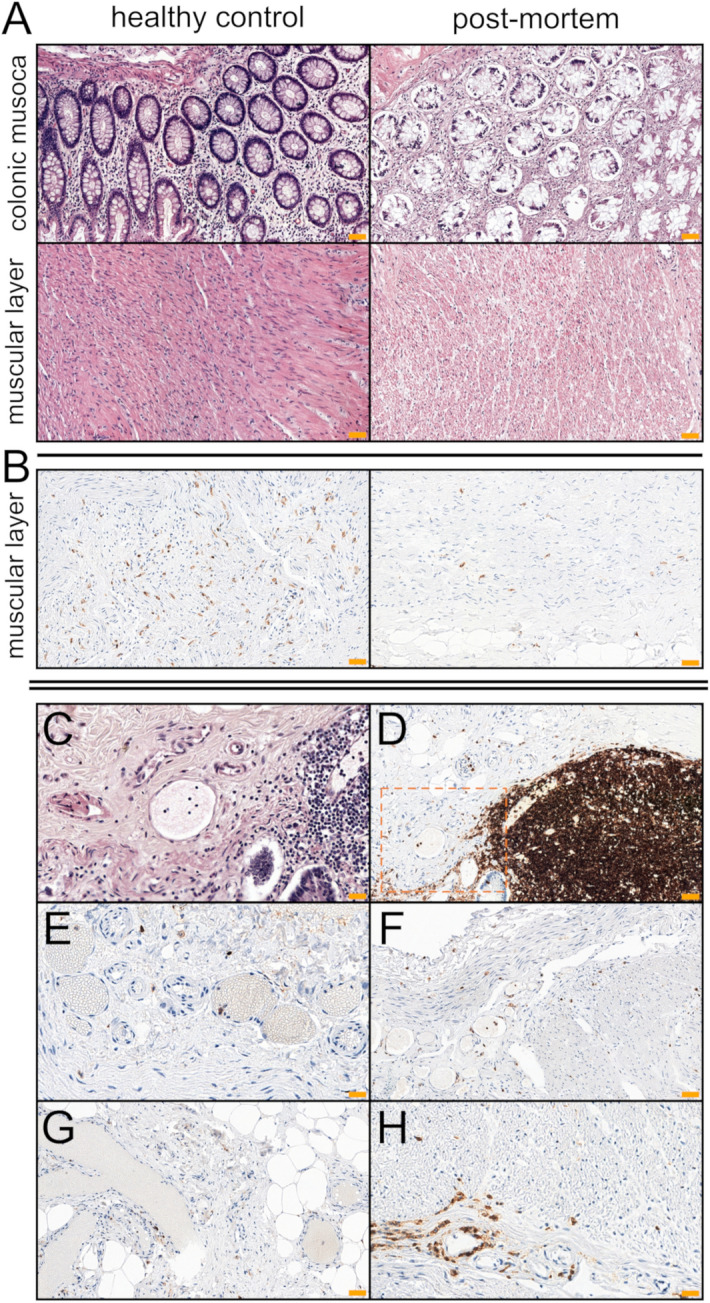
(**A**) Comparison of colonic glands and muscular layer between normal colonic mucosa (HC) and post-mortem samples (PM); H & E staining. As opposed to the normal colonic mucosa, the post-mortem samples showed discernible features of tissue autolysis, particularly pronounced within the colonic crypt. The features of early decomposition as well as slight fibrosis in the submucosal area and muscular layer were also observed. The PM cases also showed a thinner mucosa and muscularis mucosa thickness. (**B**) Mild inflammatory infiltrates (CD45^+^ cells) of the muscular layers were observed mostly in post-mortem samples. for which the starting points were most likely lymphoid clusters (**D**). The distribution of CD45^+^ cells in the post-mortem tissue was also higher in the vascular beds (**C**,**D**,**F**,**H**). In normal intestinal walls, the endovascular presence of immune cells was rarely present (**E**,**G**). The inflammatory nature of cells with vascular localization (**C**; H & E staining) was confirmed by immunohistochemical reactions against the CD45 antigen (**D**). Scale bars = 50 μm.

Healthy surgical margins obtained during colectomy which was performed in the 2nd Department of General and Oncological Surgery, Wroclaw Medical University, Poland in 2017 were used as control samples. All patients underwent surgery due to primary colorectal adenocarcinoma. From the archival database of 107 patients, 16 patients with the most similar demographic homology to the post-mortem (PM) group were selected for further studies. For that purpose, the following inclusion criteria were established: age < 69 years, male participants, no chronic comorbidities, no immunosuppressive treatment within 3 months prior to tumor resection and anatomical location of tissue specimen collection (ascending and sigmoid colon − eight cases for each segment). The exclusion criteria were as follows: age ≥ 69 years, female participants, a history of chronic comorbidities, previous radio and/or chemotherapy and the immunosuppressive therapy within 3 months prior to surgery. The comparison between both PM and healthy control (HC) cohorts was summarized in Table S1. The microanatomical comparison of colonic crypts and muscular layers in H & E staining between the representative images of PM and HC cases was shown in Fig. 1A.

### Slide preparation using a cryostat

Fresh-frozen tissue fragments were immersed in an optimal cutting temperature (OCT) compound and then slides of 7 μm were cut at − 20 °C. The obtained slides were used to perform standard topographic H & E staining. The preparations were evaluated using the Olympus BX41 microscope. Independent samples were collected and placed on MirrIR low-e microscope slides (Kevley) for further spectroscopic analysis.

### Immunohistochemistry (IHC)

IHC reaction was performed on 7-μm-thick slides from the large intestine. Initially, the slides were deparaffinized and endogenous peroxidase was blocked for 10 min. with Envision Flex Peroxidase-Blocking Reagent (Agilent, Santa Clara, USA). Mouse monoclonal antibodies (Agilent, ready-to-use antibody) against CD45 (IR75161-2), CD4 (IR64961-2) and CD8 (IR62361-2), and the rabbit polyclonal antibody against the CD3 antigen (IR50361-2) were used as primary antibodies (20 min.). Then slides were incubated in EnVision FLEX/HRP (20 min.). The reaction was visualized with freshly prepared 3,3'-diaminobenzidine (DAB) (5 min.). The slides were additionally stained with EnVision Flex Hematoxylin (5 min.). After IHC reaction and staining, the slides were dehydrated in ethanol (70%, 96%, absolute) and xylene, then closed with Dako Mounting Medium (Agilent). Further reaction was performed on Dako Autostainer Link48 (Agilent).

### Digital assessment of the whole slides

**Figure 2:**
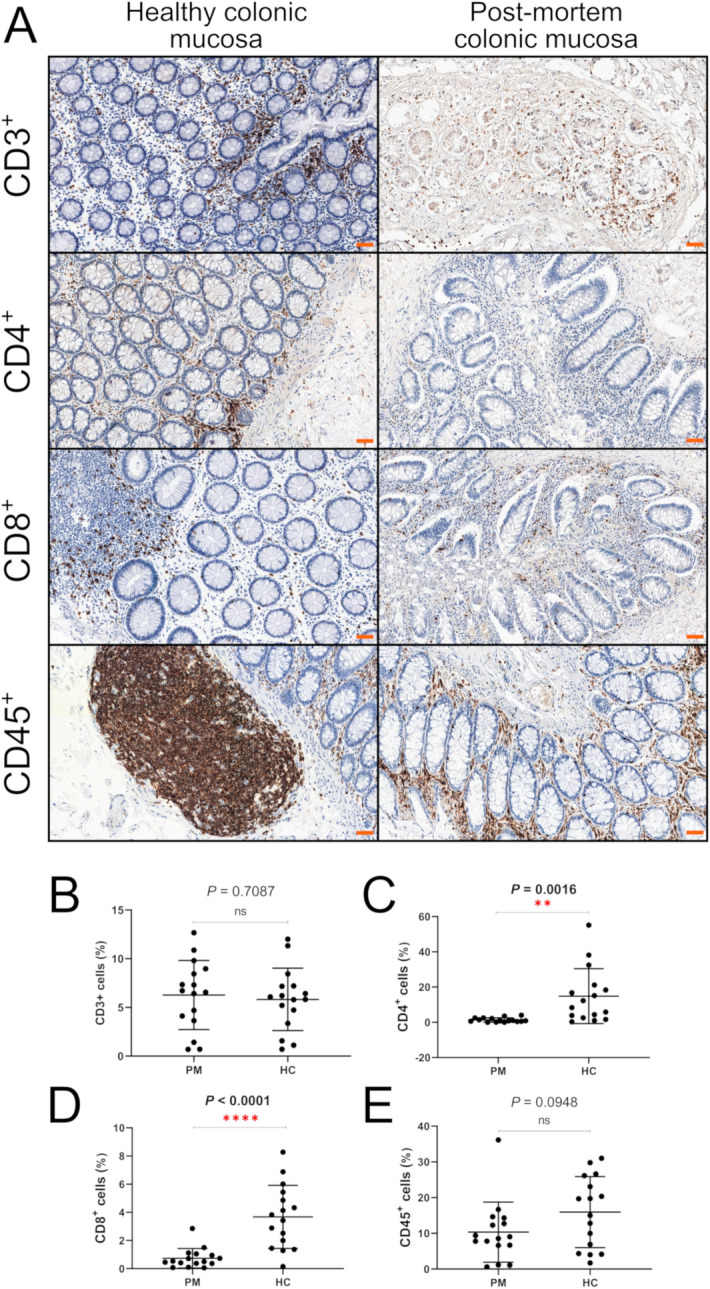
(**A**) Representative photos showing the distribution of CD3^+^, CD4^+^, CD8^+^ and CD45^+^ cells in normal and post-mortem intestinal mucosa. Particular attention should be drawn to clusters of lymphoid tissue, which seem to be the main source of immune cells infiltrating the lamina propria. Scale bars = 50 μm. (**B**−**E**) Comparison of immune cell numbers between normal colonic mucosa (HC; n = 16) and post-mortem samples (PM; n = 16).

The degree of immunohistochemical expression of the examined antigens was assessed using a digital method (QuantCenter software; 3DHistech). For this purpose, each whole slide was scanned using the Pannoramic MIDI II scanner (3DHistech) at 40 × magnification, and the resulting images were pre-prepared for further evaluation with CaseViewer (3DHistech). To determine the percentage of positive immune cells displaying membrane expression of CD45/CD3/CD4/CD8, CellQuant (3DHistech) software module was used. Representative images with the positive immunohistochemical reactions against selected antigens are presented in Fig. 2A.

### The acquisition of refractive index data

The tissue samples of 9 µm thickness on a microscope slide were flooded with phosphate-buffered saline (PBS, ThermoFisher Scientific, pH = 7.4) as the reference medium to decrease the refractive index difference and were covered by a coverslip. The refractive index (RI) of the PBS was equal to 1.3341 and was measured by the Abbe refractometer (NAR-2 T, ATAGO Co., minimum scale: 0.001) at 20 °C. The 3D RI distribution of tissue samples was obtained by limited-angle holographic tomography (LAHT) in which projections required for 3D-RI map reconstruction were registered as the series of the digital holograms of the sample, which were illuminated from different angles by the scanning beam. For this purpose, the digital holotomographic microscope (3D Cell Explorer) was used. It was based on the off-axis Mach–Zehnder interferometer with the scanning rotatable mirror. The digital holograms which were the superposition of the reference beam and the object beam scattered by the sample were registered by an imaging system consisting of an objective lens (60 × , the numerical aperture NA = 0.8, Nikon) on a digital camera. The measurements were performed in the laboratory at 22 °C.

### The processing of refractive index maps and statistical analysis

**Figure 3:**
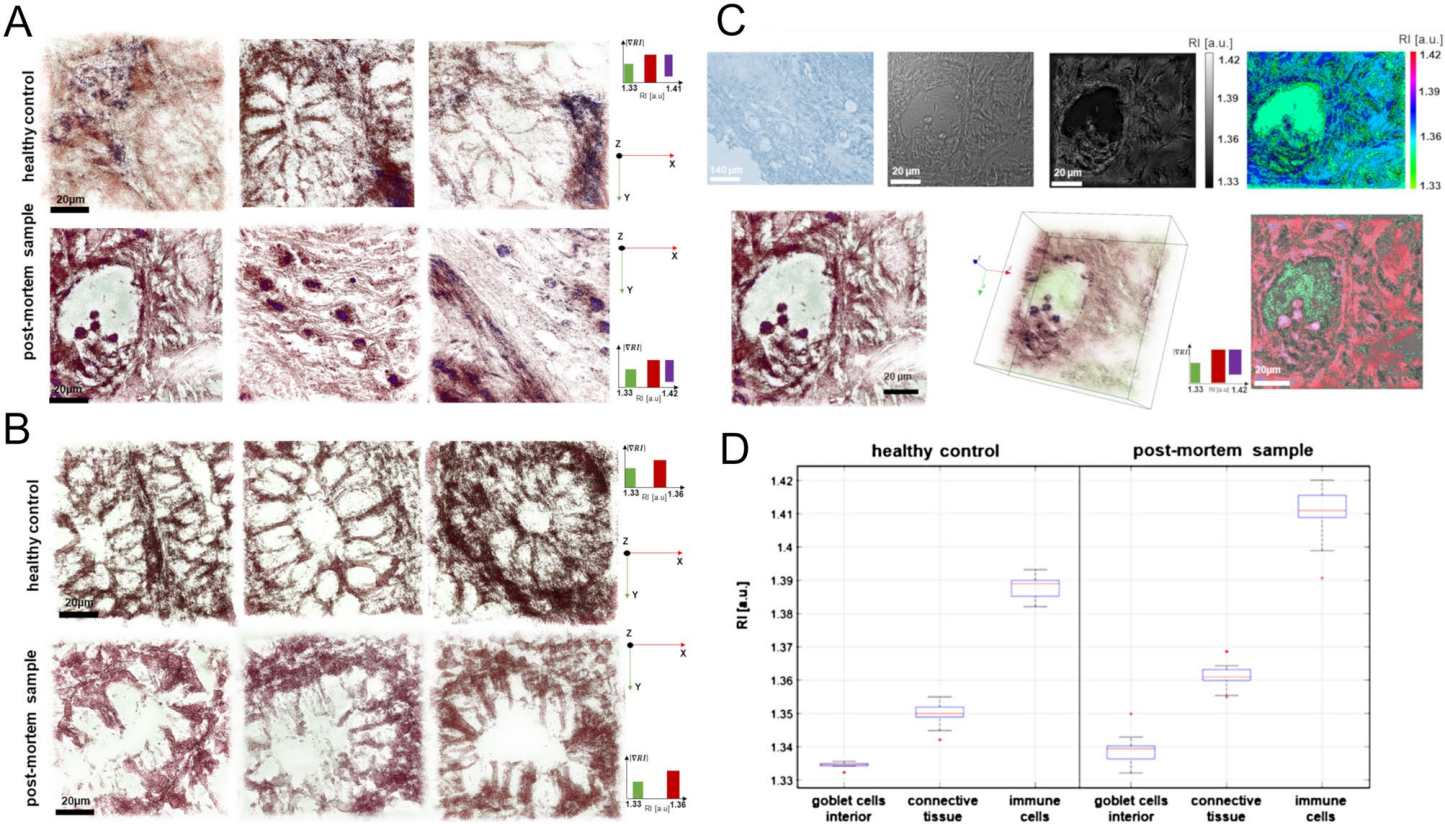
The representative results of the comparative analysis of the 3D-RI maps (upfront) of healthy control (n = 4) and post-mortem tissue samples (n = 4): (**A**) the cells with round and oval nuclei with a lymphocyte-like morphology present in the inter colonic glands and in the lumen of vessels and connective tissue, cell nuclei marked in violet; identified as immune cells (**B**) the goblet cells of colonic glands; (**C**), The schema of the RI data processing: (top row, first from the left) the microscopic image of the tissue sample, (top row, second from the left) the enlarged bright-field image of the region of interest, (top row, third from the left) reconstructed 2D-RI map, (top row, firs from the right) processed 2D-RI map, (bottom row, first from the right) digital staining of the tissue structures (green—an internal region of vessel lumen, red—connective tissue, violet—the nuclei of immune and endothelial cells), the digitally stained 3D-RI map of the tissue region (bottom row, center), the upfront of the digitally stained region of tissue area (bottom row, first from the left); (**D**) the boxplot representing the variations of the RI values of different tissue substructures (green—goblet cells interior, red—connective tissue, violet—immune cells).

The full scheme of the evaluation process for the retrieval of RI images is given in Fig. 3. At the first stage, the region of interest containing the most characteristic tissue structures was determined based on bright-field microscopy (Fig. 3C, top row, first from the left). Then, the same region of interest was used for the examination using DHT (Fig. 3C, top row, second from the left). Next, the digital holograms acquired by the DHT were reconstructed using STEVE software (Nanolive) to determine a series of 2D-RI maps (Fig. 3C, top row, third from the left). For each sample, over fifteen 3D-RI maps were reconstructed, which corresponds to 70% of the total volume of the examined tissue. Next, the 2D-RI maps (slices) in x–y planes were processed for different axial locations (Fig. 3C, top row, fourth from the left) to select the slice with the best contrast of the substructures of the tissue. Afterwards, the RI-values were used for digital staining of the selected substructures to visualize them (Fig. 3C, bottom row, first from the right). After staining, the 3D distribution of the RI of the tissue was obtained (Fig. 3C, bottom row, second from the right). All 3D visualizations were rendered. The obtained RI data enabled the quantitative and qualitative characterization of the analyzed tissue samples. For the qualitative analysis, the upfront images of the digitally stained 3D-RI maps were selected (Fig. 3C, bottom row, third from the right). In the quantitative analysis, the averaged 3D-RI values corresponding to the internal region of goblet cells, connective tissue and immune cells were determined to characterize the differences between the healthy control and post-mortem tissue samples. To examine the statistical significance of the variation of the averaged RI values of the tissue structures, the one-way analysis of variance (ANOVA) was performed. The result quantitatively indicated if there existed statistically significant differences between the means of populations. If the determined p-value for the F-statistic is smaller than the assumed significance level (in our case 0.05), the test rejects the null hypothesis that all group means are equal. The normality assumption of the extracted averaged 3D-RI values of single cells was verified by the Anderson–Darling test. Statistical analyses of the local refractive index, determination of the averaged 3D-RI values and image processing were conducted using MATLAB R2020a scripts.

### Digital identification of regions of interest (ROIs) for spectroscopic examination

**Figure 4:**
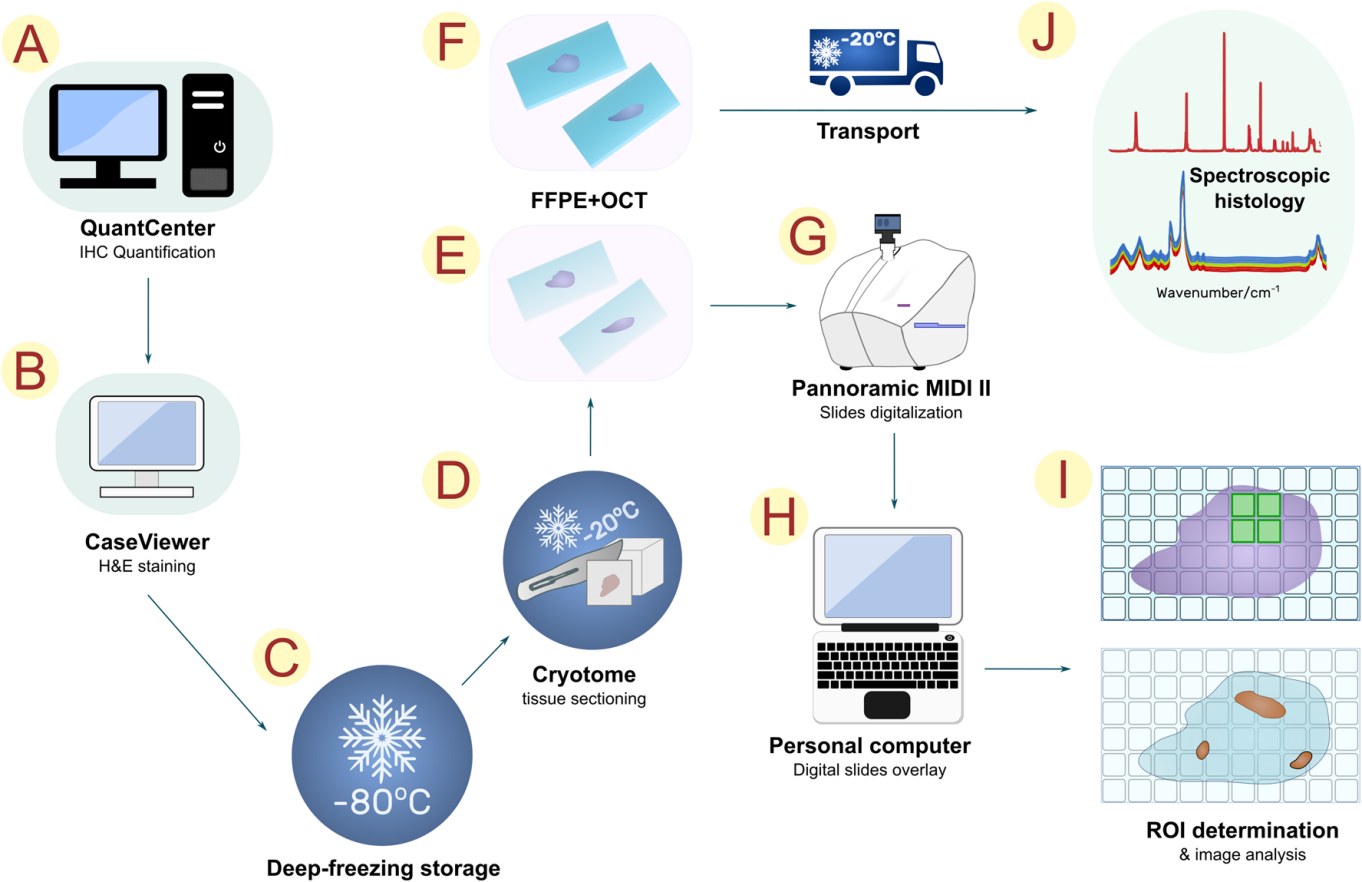
Schematic methodology for sample estimation. (**A**) Digital evaluation of formalin-fixed, paraffin-embedded (FFPE) slides of IHC expression against tested immune cell biomarkers. (**B**) Digital evaluation of homologous slides in standard hematoxylin and eosin (H & E) staining. (**CV**) Case selection of appropriate frozen tissue samples. (**D**) Tissue processing using the cryotome. (**E**) Adhesion of colon tissue sections to Superfrost glass slides for the validation of frozen tissue quality; H & E staining. (**F**) Tissue cross-sections mounted on Kevley slides for FFPE samples and frozen tissues fixed in optimal cutting temperature (OCT) compound for further spectroscopic assessment. (**G**) Scanning procedure for FFPE and H & E slides. (**H**) Selection of the most suitable areas of the colonic mucosa for spectroscopic analysis to assess GALT and inflammatory cells. (**I**) Spectroscopic examination of FFPE and OCT slides.

The most prospective cases were selected for further spectroscopic analyses based on computational assessment of positive immunohistochemical reactions for the expression of cluster of differentiation (CD) antigens identifying the immune cells. The additional criterion for sample selection was clearly preserved the intestinal wall microstructure of the large intestine with clearly marked mucous membrane. For this purpose, the whole histological slides prepared from frozen tissues in standard H & E staining were independently assessed with CaseViewer (3DHistech). Additional FTIR and Raman images were taken for the previously selected cases. To identify the most representative sites of lymphoid tissue and inflamed colonic mucosa, were applied for H & E and IHC staining. The whole histological slides representing H + E staining and IHC reactions against CD45^+^ positive cells were applied for spectroscopic imaging as new layers, for which the opacity was reduced accordingly. The most representative ROIs for the presence of immune cells were selected for spectroscopic measurements. For better clarity, a graphic diagram of the procedures is shown in Fig. 4. The overlapping of each layer to help define the ROIs is presented in Figure S1.

### Fourier transform infrared spectroscopic imaging and data analysis

An Agilent 670-IR FTIR spectrometer coupled with a 620-IR microscope was employed to acquire IR images of OCT and FFPE colon tissue cross-sections mounted on a Kevley slides. FFPE cross-sections were deparaffinized before FTIR imaging. A focal plane array (FPA) detector consists of a matrix of 16 384 pixels arranged in a 128 × 128 grid format. 15 × Cassegrain objective and condenser optics with NA of 0.62 gave a projected FPA pixel size of 5.5 μm × 5.5 μm from an area of approximately 700 μm × 700 μm. All FTIR spectra were recorded by co-adding 32 scans in the range of 900 to 3800 cm^−1^ with a spectral resolution of 4 cm^−1^.

Pre-processing of FTIR images and chemometric analysis were performed using CytoSpec (ver. 2.00.01)^29^, MatLab (R2015a, Natick, MA, USA), and Origin 9.1 (ver. 2018b, OriginLab, Northampton, MA, USA) software. Firstly, a quality test in the region between 1620 and 1680 cm^−1^ was employed to eliminate signals with absorbance lower than 0.2 and greater than 1.2. To remove spectral noise, we performed PCA-based noise reduction with 15 principal components (PCs). Resonant Mie scattering EMSC correction using seven principal components was performed on all spectra^30^. Next, second derivative IR spectra were calculated with 13 smoothing points according to the Savitzky–Golay protocol. All spectra were then vector normalized in the regions of 900–3800 cm^−1^ and 914–1770 cm^−1^ for OCT and FFPE samples, respectively, to avoid differentiation to the sample thickness. Unsupervised hierarchical cluster analysis (UHCA) was performed in the spectral regions of 1/ 970–1770 and 2800–3050 cm^−1^ for OCT samples and 2/ 970–1770 cm^−1^ for FFPE samples using second derivative FTIR spectra. Spectral distances were computed as D-values and the individual clusters were extracted according to the Ward’s algorithm. This approach groups tissue structures according to their spectral similarities and reduces hyperspectral database. Mean spectra of clusters representing chemical composition were illustrated in the form of graphs (Origin 9.1).

### Raman spectroscopic imaging and data analysis

Raman measurements of frozen OCT cross-sections of colon tissue were performed using a WITec's standard alpha300 confocal Raman imaging microscope. The spectrometer was equipped with an air-cooled solid state laser operating at 532 nm, a 600 grooves/nm grating and a CCD detector. The laser was coupled to the microscope via an optical fiber with a diameter of 50 μm. Raman images were acquired using a 100 × air objective (NA = 0.9) from a 20 μm × 20 μm ROI with a sampling density of 1 μm (in total 400 Raman spectra per image). The spectra were recorded with a spectral resolution of 3 cm^−1^ and the integration time of 2 s. Raman data pre-processing and analysis were performed using WITec software (Witec Project Plus version 5.1). Raman images were analyzed by k-Means Cluster Analysis (KMCA) using the Manhattan distance algorithm after cosmic spike removal and background correction. Mean spectra of clusters representing chemical composition were illustrated as graphs (Origin 9.1).

### Statistical analysis

Prism8 software was used for statistical analysis (Graphpad, product version: 8.4.2). Differences in the level of expression between the two test groups were assessed using an unpaired t-test. The correlation between the selected parameters was assessed using the Spearman test. The level of statistical significance was set at significant for α < 0.05 (two-tailed p value).

## Results

Immunohistochemical evaluation of T-cell markers was used for the immunophenotyping of colonic mucosa. Antigens with performing significant functions in adaptive immunity such as CD45, CD3^31^, CD4 and CD8 were selected for IHC. CD45 is a well-known marker for all leukocytes, which is expressed in hematopoietic cells, including the cells of the mononuclear phagocyte system and lymphocytes^32^. The main subset of T cells with effector functions is mostly determined by the presence of CD4 and CD8 antigens^33^. The level of expression of the above markers was assessed using a digital method. The histological slides were scanned using a Pannoramic MIDI II (3DHistech) scanner. The preparation of digital slides was performed using CaseViewer software (3DHistech). Digital analysis of reaction expression was performed using the QuantCenter software (3DHistech). To count the positive cells, CellQuant module was used. The software reported the results as positivity ranges of cell membrane signals. The area of the whole histological slides was quantified, which is consistent with the prevailing criteria for histopathological diagnostics.

### The influence of the post-mortem interval (PMI) on the expression level of CD markers in the colonic mucosa

The percentage of CD45-positive cells in post-mortem tissue showed lower mean values than in the normal control (mean PM = 10.34% mean HC = 15.96%), although this relation was not statistically significant (*P* = 0.0948). Additionally, no statistically significant differences were found for CD3 expression (*P* = 0.7087). However, significant statistical differences were demonstrated for CD4 and CD8 expression. In terms of, CD4, the level of expression of this marker was significantly decreased in post-mortem samples compared to the normal colonic mucosa (*P* = 0.0016). Even more pronounced differences were observed with respect to CD8^+^ cells, which remained with a clear predominance of control intestinal mucosa rather than in tissue samples collected from cadavers (*P* < 0.0001). A specific membrane reaction was observed for all positive cells. In post-mortem cases, the distribution of inflammatory infiltrations was increased compared with normal controls. In PM samples, mild infiltrates were also noted in the muscularis externa, while the level of inflamed mucosa was predominant in the intestinal glands. Single inflammatory cells were present in the columnar epithelium of colonic crypts, whereas the distribution of immune cells was most noted in the connective tissue of the lamina propria in normal controls. GALT was the probable source of inflammatory cells which were present in the post-mortem mucous membrane (Figs. 1D, 2A). Moreover, in post-mortem samples there was a more frequent distribution of inflammatory cells in the intestinal crypts (Fig. 2A), in the muscle layers (Fig. 1B) and in the lumen of the vessels (Fig. 1C,D,F,H). The results of IHC analyses are given in the Fig. 2B–E.

### The lack of a significant effect of PMI values on intestinal inflammation

The visual macroscopic signs of death ongoing in the early PMI were described in detail^34^, nevertheless there is still little known about the changes at the molecular level. We analyzed post-mortem samples with different PMI values. However, we did not find statistically significant differences in the expression profiles for individual inflammatory markers. Of note, negative correlations were found between PMI values and the expression of CD antigens, but they were statistically significant only for CD4^+^ cells (*r* =  − 0.542, *P* = 0.032). The exact data for all positive immune cells are shown in Table 1.

**Table 1:** A comparison of PMI values (in days) and colonic mucosal immunophenotype.

Immune cells	Total no (N = 16)	PMI ≤ 4	PMI > 4	*P* value (2-tailed)	PMI (days)	
					Spearman test	
		Mean (*SD*)	Mean (*SD*)		r	*P* value (2-tailed)
CD45^+^		13.67 (10.32)	7.014 (4.455)	0.116	− 0.326	0.217
CD3^+^		7.733 (3.606)	4.815 (3.020)	0.101	− 0.428	0.100
CD4^+^		1.848 (1.346)	0.8563 (0.9745)	0.114	− 0.542	0.032
CD8^+^		0.9175 (0.8565)	0.5625 (0.4672)	0.321	− 0.317	0.230

*PMI* post-mortem interval, *SD* standard deviation.

### The expression of CD markers and clinicopathological data

With regard to demographic parameters, we found that the age of patients did not show a significant correlation with the expression of CD antigens. No statistically significant differences were found for CD45^+^ cells in post-mortem (*r* =  − 0.449, *P* = 0.083) or normal samples (*r* = 0.028, *P* = 0.919). In terms of CD3^+^ cells, no significant influence of age was found for PM (*r* =  − 0.452, *P* = 0.08) or HC patients (*r* =  − 0.273, *P* = 0.305). Negative correlation was also noted for CD4 positive cells in post-mortem cases. However, it was not statistically significant (*r* =  − 0.423, *P* = 0.104). Additionally, the age of patients did not significantly correlate with the level of CD4 expression also in the control group (*r* = 0.107, *P* = 0.693). The number of CD8 positive cells did not significantly correlate with the age in post-mortem tissues (*r* =  − 0.167, *P* = 0.536) or in normal surgical margins (*r* = 0.101, *P* = 0.709).

No significant differences were observed in the expression of CD antigens in relation to anatomical sites, for both post-mortem patients (*P*_*CD45*_ = 0.6842, *P*_*CD3*_ = 0.2691, *P*_*CD4*_ = 0.9488, *P*_*CD8*_ = 0.8691) or the normal control (*P*_*CD45*_ = 0.6413, *P*_*CD3*_ = 0.9781, *P*_*CD4*_ = 0.8017 and *P*_*CD8*_ = 0.7983).

### The differentiation of the tissues based on the averaged 3D-RI maps

In the proposed approach, the DHT was used to reconstruct the quantitative 3D-RI maps of the tissue samples (healthy control and post-mortem) and to perform the qualitative and quantitative analyses of 3D-RI maps (see Fig. 3). For DHT method 8 histological slides were enrolled representing 4 post-mortem patients (PM) and 4 samples with healthy colonic mucosa (HC). A total number of 120 × 3D-RI tomograms, 15 for each slide, were performed. The field of view for each reconstruction was 85 × 85 µm, and a single measurement allowed to visualize the tissue area equal to 7225 µm^2^. The following number of items were analyzed for the selected slides: immune cells (HC, n = 53; PM, n = 57), intestinal glands (HC, n = 58; PM = 61) and regions of connective tissue (HC, n = 65; PM, n = 65).

The obtained results showed that this technique could be used for imaging tissue substructures as in the case of classical optical microscopy but based only on the changes in RI values related to the local variation of the density. In the case of the healthy control, it was possible to distinguish the characteristic spatial structure of the colonic glands with the goblet cells limited by a mucous membrane (lamina propria) and vessel lumen in the central regions (Fig. 3A). However, in the case of post-mortem samples, the spatial structure of the colonic glands was disrupted, which indicated a cellular decomposition process. The RI in the ‘goblet cell interior’ (the area inside goblet cells), which had values very close to the RI of the surrounding medium (1.3341), could indicate that the crypts were empty and paraffin and the preparation leached out the cell content (Fig. 3A,B). In Fig. 3A, the immune cells were visualized in the case of both types of tissue samples. In the case of the healthy control, the immune cells were located mainly in the mucous membrane (lamina propria) and connective tissue, while in the case of the post-mortem samples, they were present in the vessel lumen (Fig. 3A, bottom row, left) or connective tissue (Fig. 3A, middle/right).

The quantitative analysis based on the analysis of the 3D-RI lamps showed that there existed a significant variation of RI-values between ‘goblet cell interior’, connective tissue and immune cells (Fig. 3D). These results suggest that the DHT has the potential to be used in histopathological differentiation with high biological specificity. The highest RI values were obtained for immune cells, which indicated high differentiation accuracy of these cells by DHT. Moreover, the RI values of the tissue substructures had higher values for post-mortem samples, which indicated the differences between the spatial distribution of the tissue density, which caused the local variation of refractive indices of the analyzed substructures. To confirm our observation, the one-way ANOVA was performed. The obtained results are shown in Table 2.

**Table 2:** The results of the ANOVA, where each from 6 groups represent the set of average RI of goblet cells interior, connective tissue and immune cells for healthy control and post-mortem samples.

Source of variability	SS	df	MS	F	Prob > F
Group (between)	0.13189	5	0.02638	1816.07	4.34835 × 10^–99^
Error (within)	0.00253	294	0.00001		
Total	0.13442	299			

*SS* is a sum of squares due to each source, *df* degree of freedom associated with each source, *MS* mean squares for each source, *F* F statistics, *Prob > F* p-value which is the probability that F-statistic can take a value larger than computed F-statistic value.

Each tissue substructure (group) was characterized by 50 determined averaged RI-values. The estimated p-value for the F-statistic was significantly smaller than the significance level (0.05), which meant that the test rejected the null hypothesis that all group means were equal. Moreover, the RI variability due to the differences among the group means (variability between groups) was more significant than variability due to the differences between the data in each group and the group mean. It meant that there existed a statistically significant difference between the analyzed groups corresponding to the averaged RI values of tissue substructures, while the RI variability within groups indicated the repeatability of the determined averaged RI values of the substructures. Moreover, statistical analysis confirmed that there were also statistically significant changes in the RI values between the healthy control and post-mortem samples.

### FTIR and Raman spectroscopic imaging of healthy control and post-mortem colon cross-sections

Healthy control (HC) and post-mortem (OCT) frozen cross-sections of the colonic mucosa taken intraoperatively from healthy surgical margins were examined by FTIR and Raman imaging supported by cluster analysis (Figs. S2 and S3). The UHCA false-color map computed for the ROI scanned by using large-area FTIR imaging showed differentiation of the HC cross-section into 5 classes distributed in the intestinal wall and colonic crypts (aqua and grey), the muscular layer on the border between the wall and intestinal glands (blue), the epithelium (pink), and goblet cells with mucus (green), (see Fig. S2A). It was done by the comparison with adjacent sections stained with H & E. Codes of colors used in other IR UHCA maps of other samples indicated similar chemical composition. Clustering of a small ROI in the region of crypts only did not show the presence of additional classes suggesting that FTIR imaging of large ROIs give an insight into the full chemical information (Fig. S2C). Positions of IR bands in all spectra were assigned to biocomponents of tissues (Tab. S2)^35–42^. The HC colon cross-section was clustered due to significant differences in the spectral region specific for lipids (3100–2800 cm^−1^) and proteins (regions of amide bands: 1700–1600 [amide I], 1580–1500 [amide II] and 1350–1200 cm^−1^ [amide III]). The lipid region (3100–2800 cm^−1^) generally indicated in general that the composition of lipids is very similar whereas band intensities exhibited their various concentrations. For instance, the columnar epithelium of goblet cells (green) showed the most lipidic nature as opposed to the submucosa (blue) and the lamina propria (pink). Interestingly, esterified lipids and cholesterol (1742 cm^−1^) were accumulated in the submucosa. The protein regions indicated substantial differences in secondary conformations of proteins and their content (regions of amide bands: 1700–1600 (amide I), 1580–1500 (amide II); and 1350–1200 cm^−1^ (amide III); (c.f. Tab. S2). Here, a typical IR signature of collagens was observed for the muscular layer (blue; amide III bands: 1340, 1283, 1237, and 1203 cm^−1^) while the dominant content of α-helices in protein composition was found for the epithelium (pink, a band at 1651 cm^−1^). The wall of the crypts (green and aqua) showed the presence of a high-intensity band at 1635 cm^−1^ attributed to β-sheets. Mean IR spectra of the large intestinal glands composed of epithelial and goblet cells also indicated a high content of glycans (bands at 1060 and 1082 cm^−1^; Figs. S2C and S4, Tab. S2). The Raman image of a colonic crypt displayed in Figure S2B showed only one class and its spectrum highlighted the presence of heme proteins (1587, 752 cm^−1^).

Surprisingly, the Raman imaging of the large intestinal glands from the post-mortem sample visualized the lamina propria (aqua) and the columnar epithelium (green), see Figure S3B. The Raman spectra suggested the synthesis of unsaturated fatty acids (3010, 1658 cm^−1^) and phospholipids (1085 cm^−1^) in the post-mortem process (c.f. Figs. S3B, S5A and Tab. S2). Heme proteins observed in HC were probably absent in PM transformations. A spectral difference between the two Raman classes was related only to a higher content of lipids in the epithelium compared to the lamina propria. In turn, the IR image of post-mortem and healthy control samples was grouped in a similar number of classes—tissue structures—as for the freshly collected HC intestine (Fig. S2C and S3C). Only IR features of the columnar epithelium (green classes in Figs. S2 and S3) indicated substantial molecular changes in the PM cross-section. IR spectra confirmed the presence of unsaturated lipids as indicated by Raman spectroscopy and additionally showed a significantly increased level of esterified lipids and cholesterol. In turn, the amide regions specific for secondary structures of proteins exhibited some alterations in proteins containing β-sheet conformations since a 4 cm^−1^ red-shift of the 1635 cm^−1^ band was observed for PM (Fig. S5, Tab. S2). In addition, the occurrence of the 1681 cm^−1^ band suggested that proteins of the columnar epithelium underwent an aggregation process stabilized by intramolecular hydrogen bonding as previously reported for inclusion bodies and folding aggregates^43,44^. Chemical changes due to the post-mortem process also occurred in the region below 1150 cm^−1^ indicating transformations/formation of biomolecules containing the sugar moieties, and particularly the content of RNA increased considerably for PM (1116 cm^−1^), (c.f. Fig. S5 and Tab. S2)^42^.

### FTIR spectroscopic imaging of the intestinal mucosa with different inflammatory infiltration intensity and gut-associated lymphoid tissue

**Figure 5:**
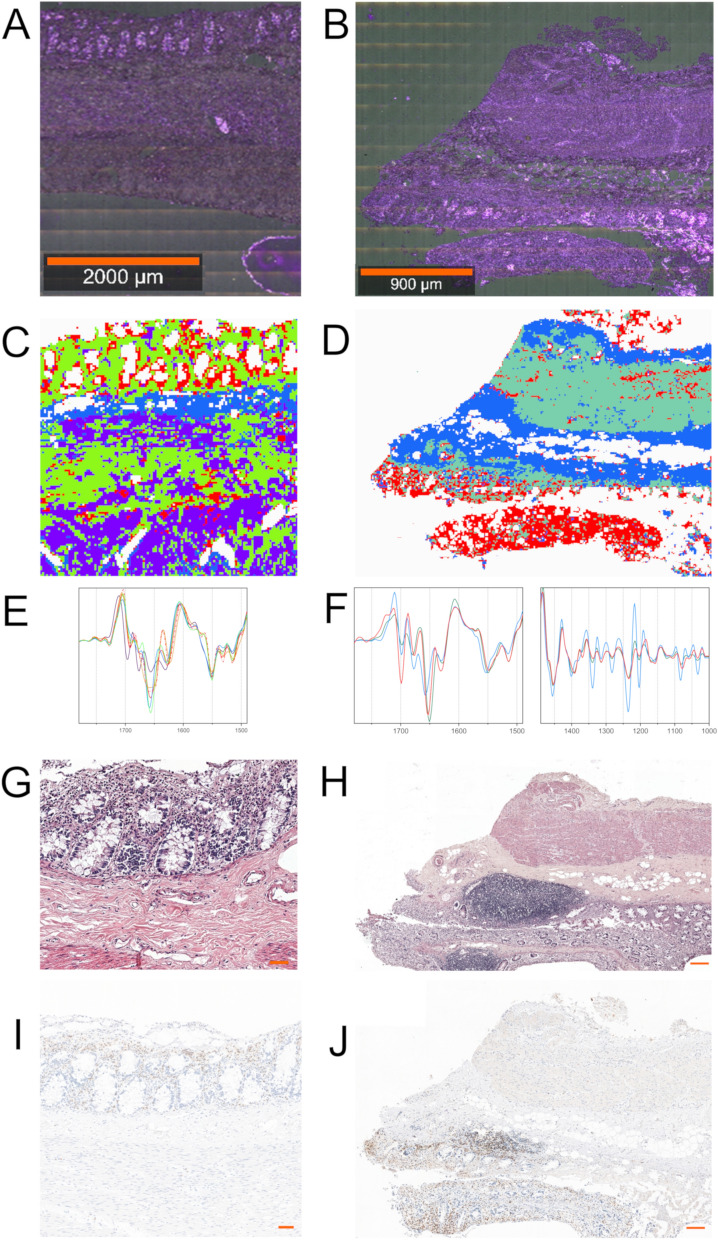
(**A**) A white-light image of post-mortem FFPE intestinal cross-sections of the sigmoid colon showing two regions of interest (ROIs) with a lower distribution of immune cells (1400 µm × 1400 µm) selected for FTIR imaging (20 × magnification); (**C**) The corresponding UHCA false-color cluster map (middle) and mean second derivative IR spectra; a red dashed trace from ROI (**E**); blue—submucosa and serosa, red—immune cells and lymphoid tissue, violet—smooth muscles, light green—lamina propria and connective tissue (down). (**B**) A white-light image of a post-mortem FFPE intestinal cross-section of the ascending colon showing ROI (2100 µm × 2800 µm) with the gut-associated lymphoid tissue (GALT) selected for FTIR imaging (upper, 20 × magnification); (**D**) UHCA false-color cluster map (middle) and their mean second derivative IR spectra (**F**). The colors of spectra correspond to the colors of classes in UHCA maps. Representative histological slides of H & E staining (**G**,**H**) and immunohistochemistry against CD3^+^ cells; scale bars = 50 μm (**G**,**I**) and 200 μm (**H**,**J**).

Post-mortem FFPE cross-sections from the sigmoid intestine in Fig. 5 were investigated by large-area FTIR imaging because the Raman signal of paraffin obscures the Raman features of tissues. In turn, due to the removal of paraffin with ethanol and xylene most lipids were removed so we did not analyze the lipid region in the FTIR spectra to avoid drawing false conclusions. Samples collected from the sigmoid colon showed the number of immune cells of low and high intensity and their UHCA maps were segregated into different classes (Fig. 5A,C,E and S6, respectively). Common spectral features were found for the purple and blue clusters assigned to smooth muscles and the submucosa/serosa, respectively. The specific amide I region in the IR spectrum of the smooth muscles indicated the presence of decomposition and/or a fibrotic process in this intestinal compartment, which resulted from the formation of unfolded proteins as indicated by the presence of bands of similar intensity at 1631, 1659, and 1676 cm^−1^ (Fig S7)^45^. On contrary the submucosa and the serosa showed the IR profile typical of collagen-rich tissue. The molecular nature of immune cells was represented by the columnar epithelium (light green in Fig. 5C and beige in Fig. S6C) as well as by immune cells and lymphoid tissue attributed to the red clusters in both cross-sections. The comparison of the light green and beige traces displayed in Fig. S7 clearly showed evident molecular differences in the entire region of the IR spectrum in terms of the distribution of immune cells. First, significant alterations in secondary conformations of proteins were found in the amide I region. The columnar epithelium with a higher number of immune cells showed a decrease in the level of α-helices (1651 cm^−1^) with the formation of aggregation of proteins stabilized by intermolecular hydrogen bonding (1622 cm^−1^), which suggests a degradation process that is different than that observed for the purple class discussed above. Although, the direct vicinity of immune cells and lymphoid tissue was completely different, colonic mucosa regions of low and high number of immune cells were chemically similar, compared to the red traces (Fig. S7A).

IR results obtained for the GALT found in the ascending colon showed the molecular composition distributed in the layers assigned to muscularis mucosa and muscularis externa (blue and mint) and inflamed colonic mucosa (red), (c.f. Fig. 5B,D). No features of autolysis were observed in the IR spectra of clusters (Fig. S7B). The blue class showed the typical collagen signature whereas the red and mint clusters co-localized close to each other shared a similar profile in the spectral region below 1400 cm^−1^ (Fig. S7B). We observed the presence of glutamate amino acid (1392 cm^−1^), glycosylated proteins (1157 cm^−1^), and mucin (1106 cm^−1^) in the colonic mucosa^36^. The most pronounced difference between the mint and red classes was found in the region of the amide I bands in that an increase of absorbances specific for anti-parallel β-sheets (1699, 1633 cm^−1^) occurred in immune cells. Of note, IR signature of GALT differed significantly from immune cells in the intestinal mucosa of the sigmoid colon (Figs. S7A and S7B).

### FTIR spectroscopic imaging of post-mortem colonic mucosa with similar distribution of immune cells taken from one patient from different anatomical locations

**Figure 6:**
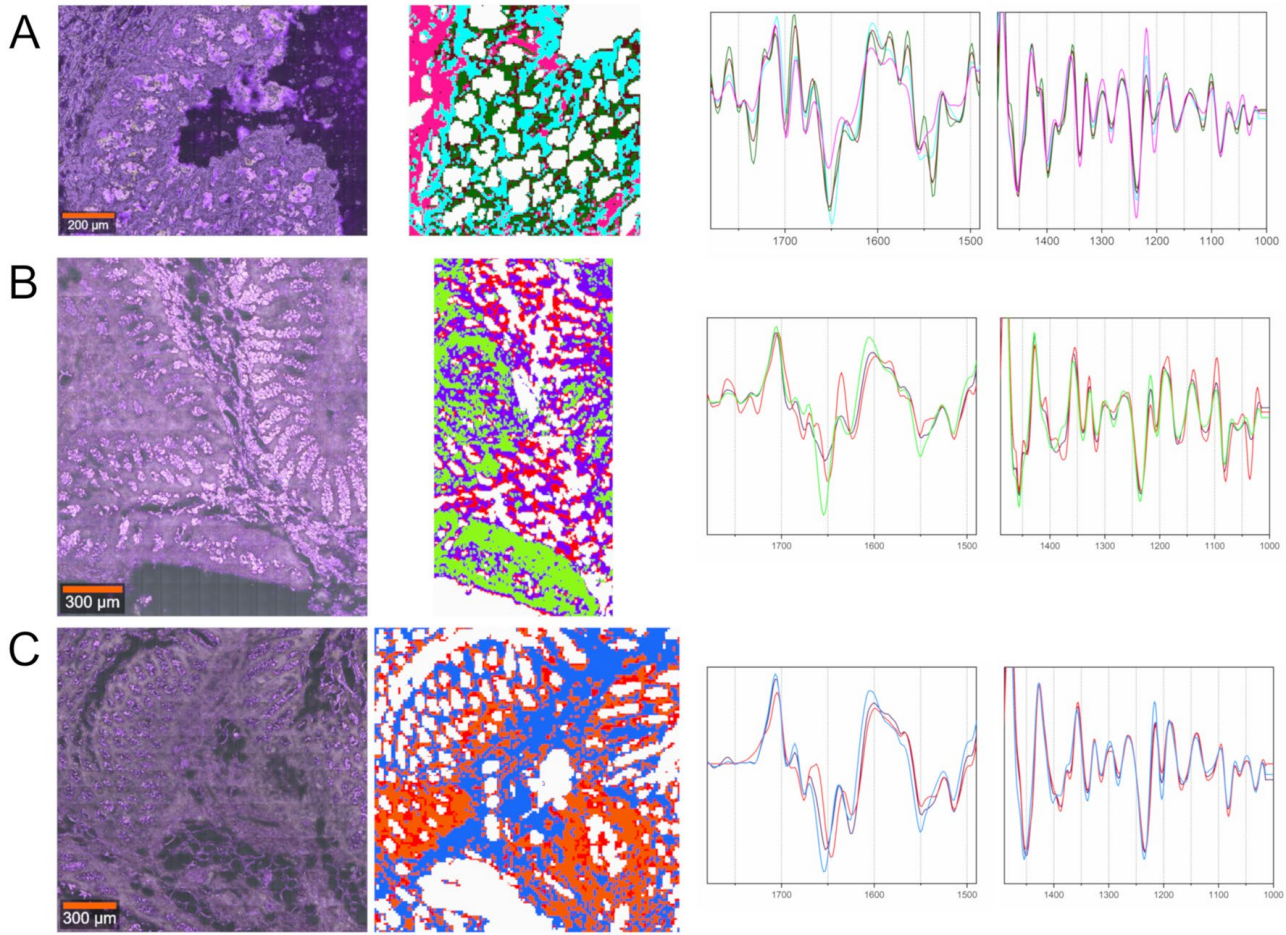
FTIR imaging of post-mortem cross-sections of frozen optimal cutting temperature (OCT) compound sigmoid colon (**A**), formalin-fixed, paraffin-embedded (FFPE) sigmoid colon (**B**) and FFPE ascending colon (**C**); (left) A: white-light images of selected regions of interest (ROIs) (**A**: 700 µm × 700 µm; **B**: 1400 µm × 700 µm; **C**: 1400 µm × 1400 µm; 20 × magnification); (middle) the corresponding UHCA cluster maps and (right) their mean FTIR spectra; green/brown—epithelial component of intestinal glands, red/orange—inflammatory cells, pink—connective tissue of the lamina propria, light green—lamina propria, aqua/purple/blue—lamina propria. The colors of spectra correspond to the colors of classes in UHCA maps.

Figure 6 shows the comparison of the results of FTIR imaging of post-mortem colonic mucosa with similar intensity of immune cells. The aim of this part of our study was to investigate of the effect of the sample preparation and anatomic localization of inflammation on its spectroscopic recognition and characterization. Cluster analysis of FTIR images of the studied three cross-sections discriminated lamina propria, the epithelium of intestinal glands, and immune cells (Fig. 6A–C). Different colors assigned to the same tissue structures indicated that their IR spectra showed molecular differences (Fig. 6 and S8). First, we compared spectral data obtained for the cross-sections of the sigmoid colon immersed in OCT and frozen and immersed in paraffin, (c.f. Fig. 6A,B), respectively. As in the case of the frozen (OCT) samples collected for HC and PM (Figs. S2 and S3), FTIR spectra of the frozen samples showed the accumulation of triglycerides in the mucosa that disappeared in the FFPE cross-sections. The connective tissue of the lamina propria, (pink and light green classes in Fig. 6A,B, respectively), showed the IR profile of collagens based on the highest intensities of amide III bands of these fibrous proteins (1340, 1283, 1237, and 1203 cm^−1^) but the amide I features strongly depended on the sample preparation (1500–1700 cm^−1^), (see Fig. S8). The similar trend of alternation of secondary conformations of proteins was found for samples shown in Figures S2 and 5. Next, the comparison of the UHCA maps and their IR spectra for the FFPE cross-section from the sigmoid and ascending colon showed autolysis and fibrotic tissue in the case the sigmoid colon (purple), (c.f. Fig. 6B,C and S8B, C). The connective tissue was similar (light green and blue in Fig. 6B,C, respectively), whereas immune cells coded as red and orange classes in the sigmoid and ascending colon, respectively, showed the presence of the IR bands specific for amyloid proteins with strong intermolecular bonding and inflamed colonic mucosa (Figs. S8B and S8C). The two classes differed in terms of an increased absorbance of the 1030 cm^−1^ band attributed to glucose in the ascending colon^46^.

## Discussion

Despite significant progress in digital pathology that has been made for decades, histopathological diagnosis continues to be a major clinical challenge and requires an interdisciplinary approach to be optimal^13^. The assessment of spatial frequency data of histological images allows the quantification of inflammatory parameters and improves evaluation of inflammation in the colon, which in relation to digital pathology can contribute to a more accurate diagnosis and can more accurately differentiate the immune cells^47^.

In our study, we successfully used selected spectroscopic methods to evaluate immune cells and lymphatic aggregates in the colonic mucosa as a methodological support for routine IHC staining. Furthermore, to increase the diagnostic value of the obtained results positive reactions against CD antigens were evaluated using a digital method in whole histological slides. Whole slide imaging, which is a recognized system of interpreting digital slides to make the initial diagnosis and is becoming more widely used^48^. The use of FTIR imaging is an innovative methodological solution to the detection of immune cells present in the examined tissue. To the best of our knowledge, the first use of FTIR imaging in differentiating immune cells for their immunophenotype was reported in 2015^49^. In our study, the FTIR imaging was used for the first time to detect GALT and immune cells in the colonic mucosa. The evaluation of molecular parameters of the colonic mucosa by RS is an additional advantage of our research. In our opinion, this is a useful method for ROI characterization of mucous membranes with different distribution of immune cells. Both spectroscopic methods may be important supportive implementation for routine staining commonly used in clinical histopathology since their results showed different spectral profiles in each case and indicated that not only proteins contributed to this discrimination. Intensities of lipids and sugar moiety signals also altered in the intestinal glands and could be used to assess tissue transformations in PM cells and the formation of inflammatory cells. Further model studies should be undertaken to propose spectroscopic technology for quantitative and/or automated analysis of IR and RS images.

Our research also provides valuable data on forensic pathology in relation to the immune component of the intestinal mucosa in post-mortem conditions. This type of analysis was also conducted for the first time. It should be noted that post-mortem samples are increasingly used in scientific research that focuses on the molecular features of cancer. The alterations ongoing in this type of tissue, which were found in our study, suggest that the use of the tissue in research on tumor immunity may not be the best solution. The conclusions of our analysis suggest that PMI has a suppressive effect on the effector properties of cell-mediated immunity. Decreased expression of CD4 and CD8 antigens supports this conclusion. As yet, no similar scientific studies have been conducted. Nevertheless, in the previous study, Sneeboer et al. showed that residual inflammatory cells such as microglia in the post-mortem brain showed no immune activity^50^, which is in line with our conclusions.

Our research has also some limitations. Despite great efforts regarding the selection of the post-mortem samples and the control group with the highest possible homology, we encountered some difficulties. One of them was connected with conducting research on one sex only. An additional limitation is related to the higher age of the control group compared to post-mortem cases. Our normal controls were comprised of healthy surgical margins taken from patients diagnosed with primary colorectal adenocarcinoma. In our database, this patient population was characterized by substantially higher age than post-mortem patients. It should be noted that the resulting discrepancies are due to the accepted criteria for including post-mortem patients in the study. During enrollment, we focused on somatically healthy patients, whose sudden deaths were mostly related to traffic accidents. The characteristics of our post-mortem human cadavers corresponded to international reports, which found that men aged 25–65 years were usually the victims of such accidents^51^. There was a risk that higher age of the control group would have an adverse effect on the expression level of the examined biomarkers. During the aging process, the function and accumulation of T cells is disturbed. Changes in the distribution of CD4^+^ T cells in the secondary lymphoid organs in which GALT is present, occur particularly in tissue compartments such as the colon and small intestinal lamina propria^52^. The results of our research, however, found no statistically significant correlations between the expression of CD antigens and the age of examined patients.

Another limitation was related to different PMI values for individual post-mortem patients included in the study. In our patients, PMI values were found between the second and the seventh day, which corresponds to the early period of decomposition^34,53^. These PMI values in forensic pathology are the norm and are considered diagnostic. In addition, all tissues were collected in the summer, which significantly reduced the adverse impact of environmental factors on the obtained results, such as air humidity and high temperature. Human cadavers were also stored under constant cooling conditions until tissue collection. During the autopsy, it was also found that the bodies and organs did not show any features of decomposition or tissue degradation and the microscopic image of tissues in standard H & E staining did not show any rotting or advanced autolysis. The study found that the PMI value did not affect the number of immune cells in the mucous membrane of the large intestine (Table 1). Our experience shows that the speed of decomposition is an individual characteristic and apart from external factors, the individual conditions also play a significant role. Therefore, a thorough examination of the body at the macro- and microscopic scale should be performed in each case, regardless of the PMI value.

In our study, we also collected tissue material from two different anatomical locations, i.e., the ascending and sigmoid colon, which was due to two reasons. Firstly, the impact of anatomical location on the analyzed molecular and cellular parameters could not be excluded^54^.

Secondly, significant changes in gene expression occur in the colon in post-mortem conditions already at a relatively short PMI. Nevertheless, the number of genes undergoing abnormal transcription varies significantly with respect to anatomical location. Therefore, the most expressed alterations were related to the transverse colon and much smaller differences were observed in relation to the sigmoid colon^55^. In our research, we did not assess the gene expression. However, the results obtained by Ferreira et al. led to the conclusion that the sigmoid colon would be the most optimal region for tissue collection in our research. In addition, both locations were most consistent with our control group.

Based on our forensic experience, there is no doubt that dehydration processes that occur in post-mortem samples certainly depend on the PMI. They are also related to the conditions in which the body is found. The post-mortem drying up in any form of the processes that lead to water loss by cells and tissues occurs differently in the fully preserved post-mortem human body and at the moment of tissue biopsy sampling. This is due to the preserved organ integrity as opposed to the sections taken during autopsies, which are usually fixed in a short time after collection. These processes leading to the cells’ and tissues’ dehydration may affect also 3D-RI distributions, what was already reported in case of the digital holographic microscopy^56^. Therefore, during the examination of the tissue’s samples by digital holographic tomography apart of the tissue fixation^21^ also the PMI should be taken into careful consideration as a parameter affecting the reconstructed RI-values of analyzed samples.

Immunohistochemistry as the medical procedure has for years brought an unquestionable diagnostic value in the field of pathology at relatively low costs, which is particularly important in view of the significant benefits resulting from the high efficiency of this method and its positive impact on the predictable survival of the studied population^57^. The use of immunohistochemical staining to identify histopathological and molecular subtypes of cancer also allows for better planning of the optimal systemic treatment for an individual patient^58^. The predictive value particularly relates to the immunohistochemical examination of expression level of tumor infiltrating-lymphocytes (TILs) biomarkers^59^. Nevertheless, it is difficult to receive clinically useful information since measuring the level of one or several biomarkers, hence the future of personalized medicine is the collective assessment of numerous integrations between various molecular data^60^, and new technological applications may significantly improve the usefulness of current diagnostic standards. The spectroscopic and DHT methods used in this study made it possible to show immune cells on the examined histological slides and offer the possibility to broaden the molecular phenotyping of examined tissues. The DHT method, by measuring the refractive index, can also be used in forensic pathology to better determine the associations between the parameters of the assessed samples and the cause of death, or the influence of the PMI value on post-mortem processes occurring in individual organs. These conclusions should be discussed in further studies on a larger cohort of cases.

## Supplementary Information

Supplementary Information.
