# Supplementary material for: Molecular profiling of the intestinal mucosa and immune cells of the colon by multi-parametric histological techniques
Source: Sci Rep. 2021 May 28;11:11309. doi: 10.1038/s41598-021-90761-y (PMC8163794; doi:10.1038/s41598-021-90761-y)
Supplement: Supplementary file 1 — Supplementary Information. [file 41598_2021_90761_MOESM1_ESM.pdf]

SUPPORTING INFORMATION

Molecular profiling of the intestinal mucosa and immune cells of the colon by multi-parametric histological techniques

Łukasz Zadka[1], Karolina Chrabaszcz[2], Igor Buzalewicz[3], Ewelina Wiercigroch[2], Natalia Glatzel-Plucińska[1], Łukasz Szleszkowski[4], Agnieszka Gomułkiewicz[1], Aleksandra Piotrowska[1], Krzysztof Kurnol[1,5], Piotr Dzięgiel[1], Tomasz Jurek[4], Kamilla Malek[2].

[1]Department of Human Morphology and Embryology, Histology and Embryology Division, Wroclaw Medical University, Wroclaw, Poland

[2]Faculty of Chemistry, Jagiellonian University in Krakow, Krakow, Poland.

[3]Bio-Optics Group, Department of Biomedical Engineering, Wroclaw University of Science and Technology, Wroclaw, Poland.

[4]Department of Forensic Medicine, Forensic Medicine Unit, Wroclaw Medical University, Wroclaw, Poland.

[5]2nd Department of General and Oncological Surgery, Wroclaw Medical University, Wrocław, Poland

TABLE S1. Characteristics of the studied groups

Case#	Specimen type	Sex	Age	Characteristics of the sample			Technique for tissue assessment				PMI (days)
				FFPE	Frozen	Anatomical Site	IHC	IR	RS	DHT	
1	PM	M	38	+		Ascending colon	+			+	7
2	PM	M	38	+	+	Sigmoid colon	+				7
3	PM	M	44	+		Ascending colon	+	+			5
4	PM	M	44	+	+	Sigmoid colon	+	+	+		5
5	PM	M	19	+		Ascending colon	+			+	3
6	PM	M	19	+	+	Sigmoid colon	+				3
7	PM	M	27	+		Ascending colon	+				3
8	PM	M	27	+	+	Sigmoid colon	+				3
9	PM	M	61	+		Ascending colon	+	+			7
10	PM	M	61	+	+	Sigmoid colon	+	+			7
11	PM	M	43	+		Ascending colon	+			+	6
12	PM	M	43	+	+	Sigmoid colon	+	+		+	6
13	PM	M	20	+		Ascending colon	+				4
14	PM	M	20	+	+	Sigmoid colon	+				4
15	PM	M	20	+		Ascending colon	+				2
16	PM	M	20	+	+	Sigmoid colon	+				2
17	HC	M	67	+	+	Ascending colon	+				-
18	HC	M	65	+	+	Ascending colon	+				-
19	HC	M	68	+	+	Ascending colon	+				-
20	HC	M	65	+	+	Sigmoid colon	+				-
21	HC	M	64	+	+	Sigmoid colon	+				-
22	HC	M	64	+	+	Sigmoid colon	+			+	-
23	HC	M	64	+	+	Sigmoid colon	+				-
24	HC	M	54	+	+	Sigmoid colon	+				-
25	HC	M	67	+	+	Ascending colon	+				-
26	HC	M	68	+	+	Sigmoid colon	+				-
27	HC	M	59	+	+	Sigmoid colon	+				-
28	HC	M	60	+	+	Ascending colon	+			+	-
29	HC	M	63	+	+	Ascending colon	+			+	-
30	HC	M	67	+	+	Ascending colon	+				-
31	HC	M	55	+	+	Ascending colon	+				-
32	HC	M	58	+	+	Sigmoid colon	+	+	+	+	-

PM indicates post-mortem; HC, healthy control; M, male; FFPE, formalin-fixed paraffin-embedded; IHC, immunohistochemistry; IR, Infrared; RS, Raman spectroscopy; DHT, Digital Holotomography; PMI, post-mortem interval.

Table S2. Positions of Infrared (IR) and Raman bands with their assignment to biocomponents[35-48]

IR / cm^{-1}	Raman / cm^{-1}	Assignment
3011, 3007	3011	stretching vibration of the =CH groups: unsaturated alkyl chains in lipids
2960	-	asymmetric stretching vibrations of the -CH_3 groups: proteins (mainly) and lipids
	2932	stretching vibrations of the -CH_3 groups: proteins (mainly) and lipids
2923	-	asymmetric stretching vibrations of the -CH_2 groups: lipids (mainly) and proteins
-	2903	Symmetric stretching vibrations of the -CH_3 groups: fatty acids
-	2878	Stretching vibration of CH_2: lipids (mainly) and proteins
2874	-	symmetric stretching vibrations of the -CH_3 groups: proteins (mainly) and lipids
2852	-	symmetric stretching vibrations of the -CH_2 groups: lipids (mainly) and proteins
-	2857	Asymmetric stretching vibrations of the =CH_2 groups: fatty acids
1742	-	Stretching vibration of C=O: triacylglycerols, glycerophospholipids
1736	-	Stretching vibration of C=O: cholesterol esters
1720	-	Stretching vibration of C=O: free fatty acids
1699	-	Amide I: antiparallel β-sheet conformation in proteins
1681,1678	-	Amide I: turns, loops in proteins
-	1658	Stretching vibrations of C=C: unsaturated alkyl chains in lipids
1655,1651	-	Amide I; α-helices in proteins
1635,1633,1631	-	Amide I: β-sheets in proteins
1622	-	Proteins stabilized by hydrogen bonding
1589	-	Asymmetric stretching vibrations of COO^-; free amino acids, free fatty acids
	1587	Asymmetric stretching vibrations of C-C: heme proteins
1557	-	Amide II: proteins
1545	-	Amide II: proteins
1514	-	In-plane deformation vibration C-H of phenyl ring: tyrosine residues
1475	-	Deformation vibration of CH_2, CH_3: proteins, lipids
1468,1458,1453	1453	
-	1445	Deformation vibration of -CH_2: lipids
1401	-	symmetric stretching vibrations of COO^-: free amino acids, free fatty acids
1392	-	Glutamine amino acids
1340<br>1314	-	Amide III: proteins
-	1310	Stretching vibrations of C-N: heme proteins; deformation of CH_2: lipids
1283	-	Amide III: fibrous proteins (mainly collagen)
1237	-	asymmetric stretching vibrations of PO_2^- group: phospholipids, nucleic acids, etc.; Amide III: fibrous proteins (mainly collagen)
1203	-	Amide III: fibrous proteins (mainly collagen)
1190	-	
1170,1167,1160	-	symmetric stretching vibrations of -CO-O-C: cholesterol esters, phospholipids, carbohydrates
1116	-	Stretching vibrations of C-O of ribose ring: RNA
1157	-	Glycosylated proteins
1106	-	Mucin
1060	-	Stretching vibrations of -CO-O-C; cholesterol esters, carbohydrates
1030	-	Deformation vibration of C-OH: carbohydrates
-	1006	Symmetric ring breathing: phenyloalanine
-	752	Ring breathing: heme proteins

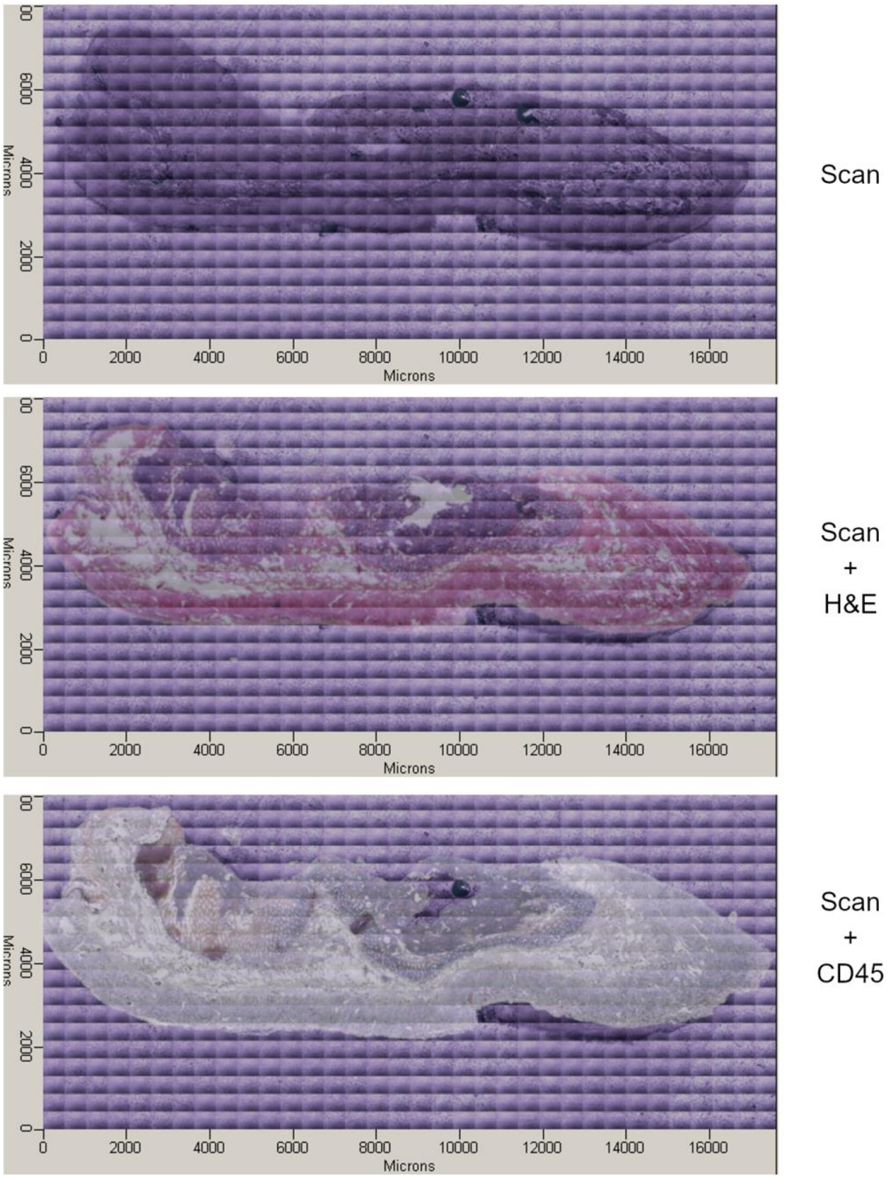

FIGURE S1. Example of superimposed microphotographs to identify regions of interest (ROIs) for spectroscopic imaging. Abbreviations: H & E, hematoxylin and eosin staining; CD45, immunohistochemical reactions against $CD45^{+}$ immune cells.

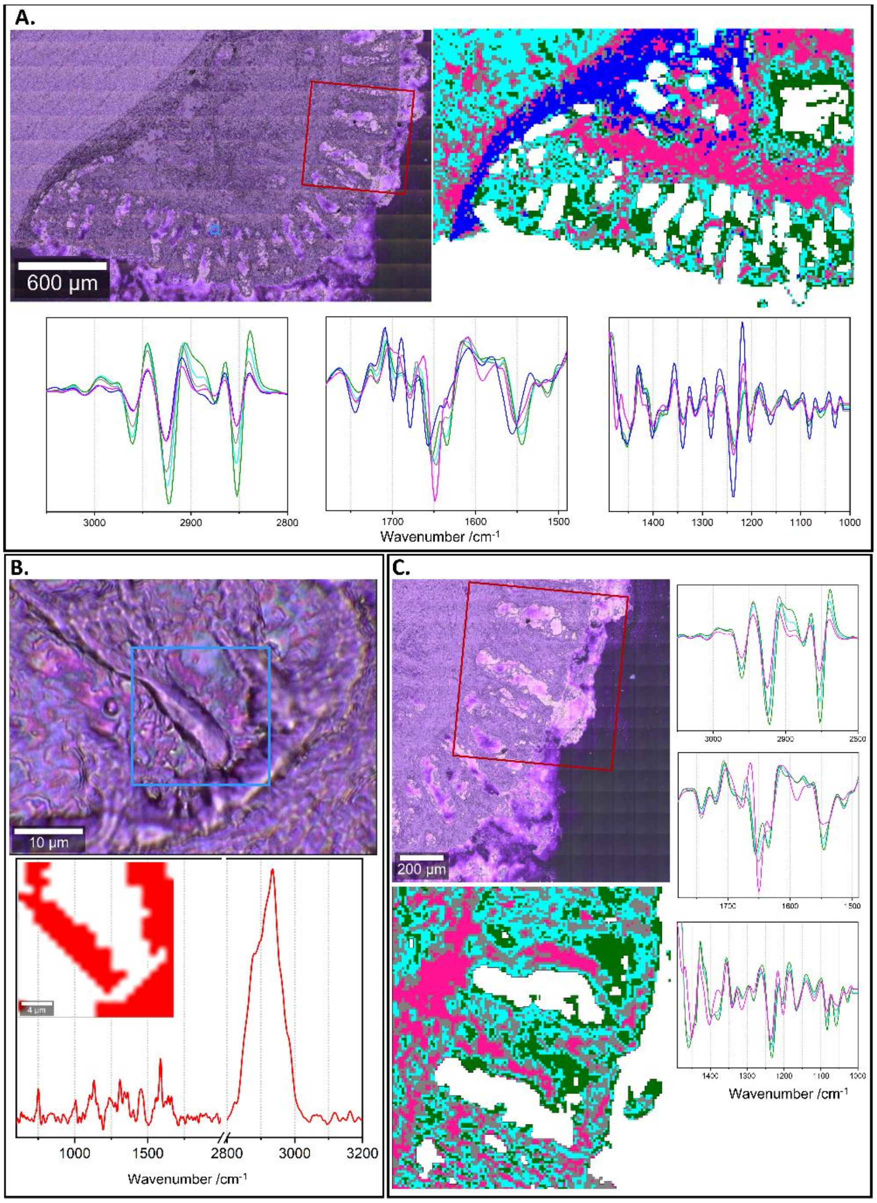

FIGURE S2. A, (left) A white-light image (20× magnification) of a frozen (OCT) cross-section of normal intestinal wall showing ROI (1400 µm × 2100 µm) selected for FTIR imaging; UHCA false-color cluster map (Unsupervised Hierarchical Cluster Analysis, right) and mean second derivative IR spectra of five classes: blue- submucosa, pink- lamina propria, green – columnar epithelium, aqua and gray- muscularis externa and lamina propria (down). B, A white-light image (100× magnification, upper) with ROI (blue square showing a colonic crypt; see its localization in A) selected for Raman imaging (20 µm × 20 µm); KMCA false-color cluster map (k-Means Cluster Analysis, inset) and mean Raman spectrum (down). C, (left) A white-light image (20× magnification) with ROI (red square showing the large intestinal glands composed of the epithelium and goblet cells; see its localization in A) selected for FTIR imaging (700 µm × 700 µm); UHCA false-color cluster map (down) and mean second derivative IR spectra of four classes (their assignment to tissue structures as in A). The colors of spectra correspond to the colors of classes in the UHCA and KMCA maps.

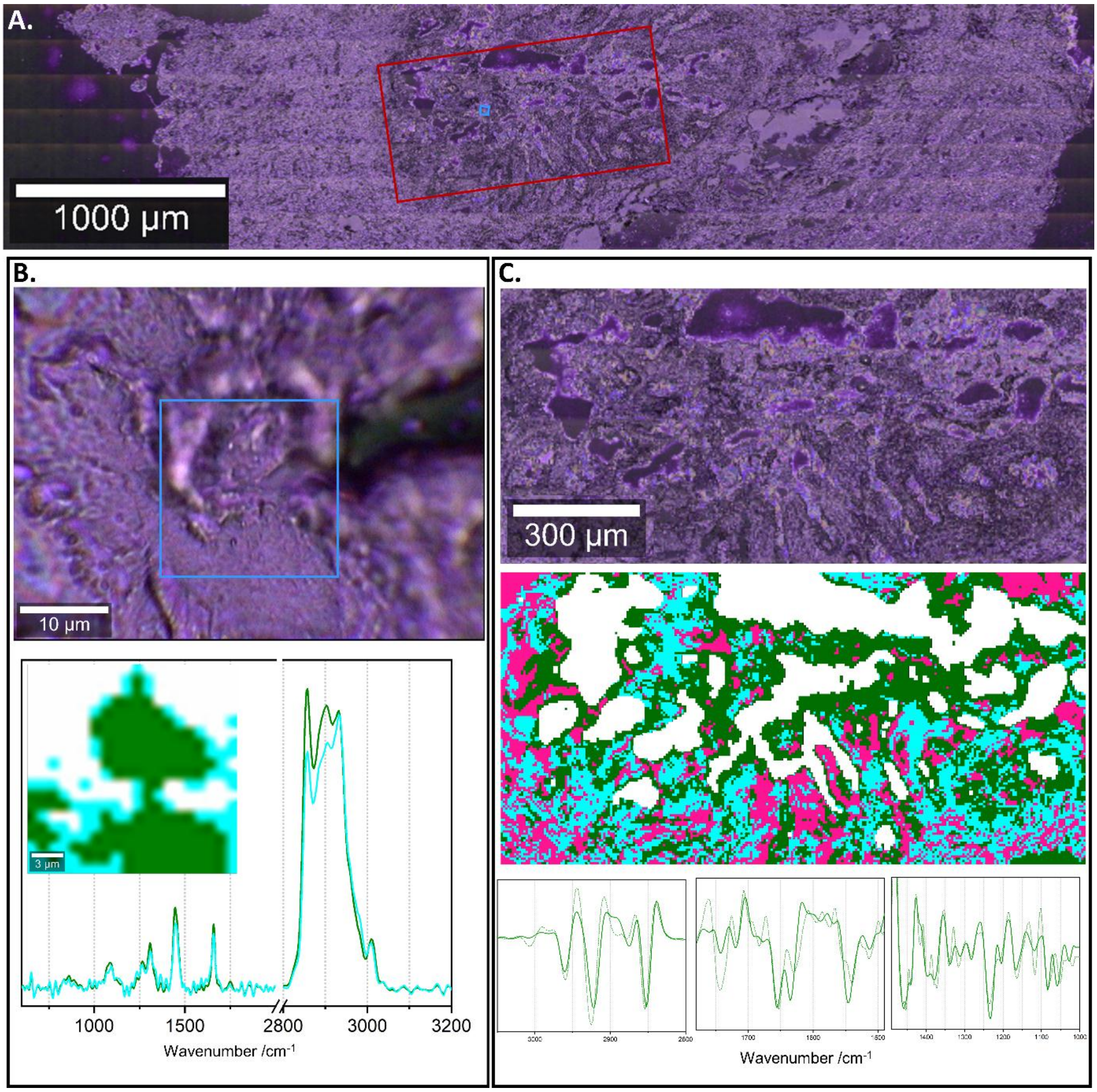

FIGURE S3. A, A white-light image (20× magnification) of post-mortem frozen (OCT) cross-section of the control large intestine with ROIs selected for Raman (blue square; 20 µm × 20 µm) and FTIR imaging (red rectangular; 700 µm × 1400 µm). B, A white-light image (100× magnification, upper) of the Raman ROI (blue square, see its localization in A) with KMCA false-color cluster map (inset) showing the lamina propria (aqua) and columnar epithelium (green) and their mean Raman spectra (down). C, A white-light image (20× magnification, upper) of the FTIR ROI (red rectangular; see its localization in A) with UHCA false-color cluster map (middle) showing the large intestinal glands composed of the epithelium and goblet cells, and mean second derivative IR spectra of the intestinal epithelium from post-mortem (green dashed trace) and control samples (solid trace from the green class in Fig. S2). The colors of spectra correspond to the colors of the classes in UHCA and KMC maps.

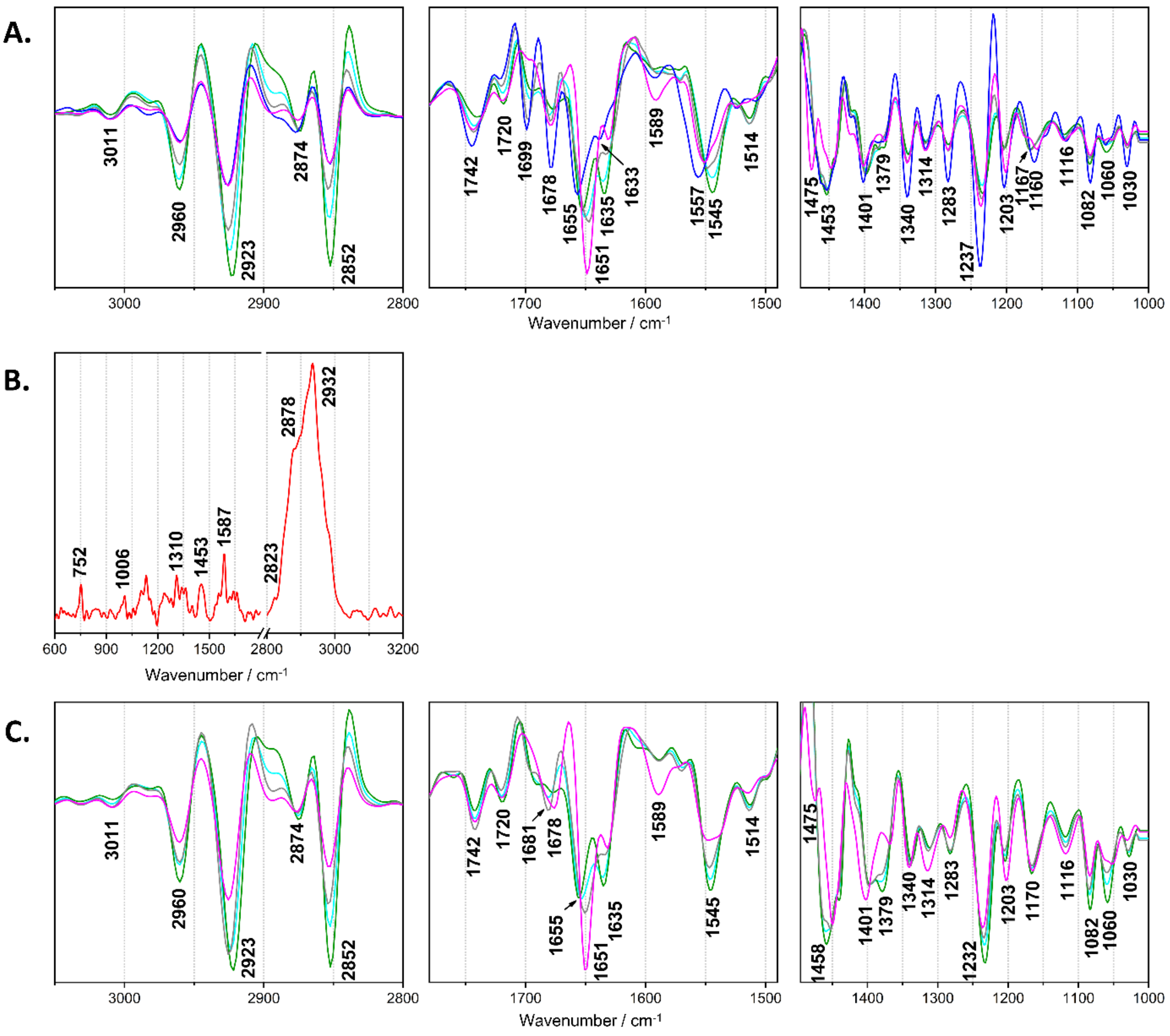

FIGURE S4. Mean FTIR (A and C) and Raman (B) spectra with band positions extracted from UHCA and KMCA analyses are given in Figure S2 A-C, respectively.

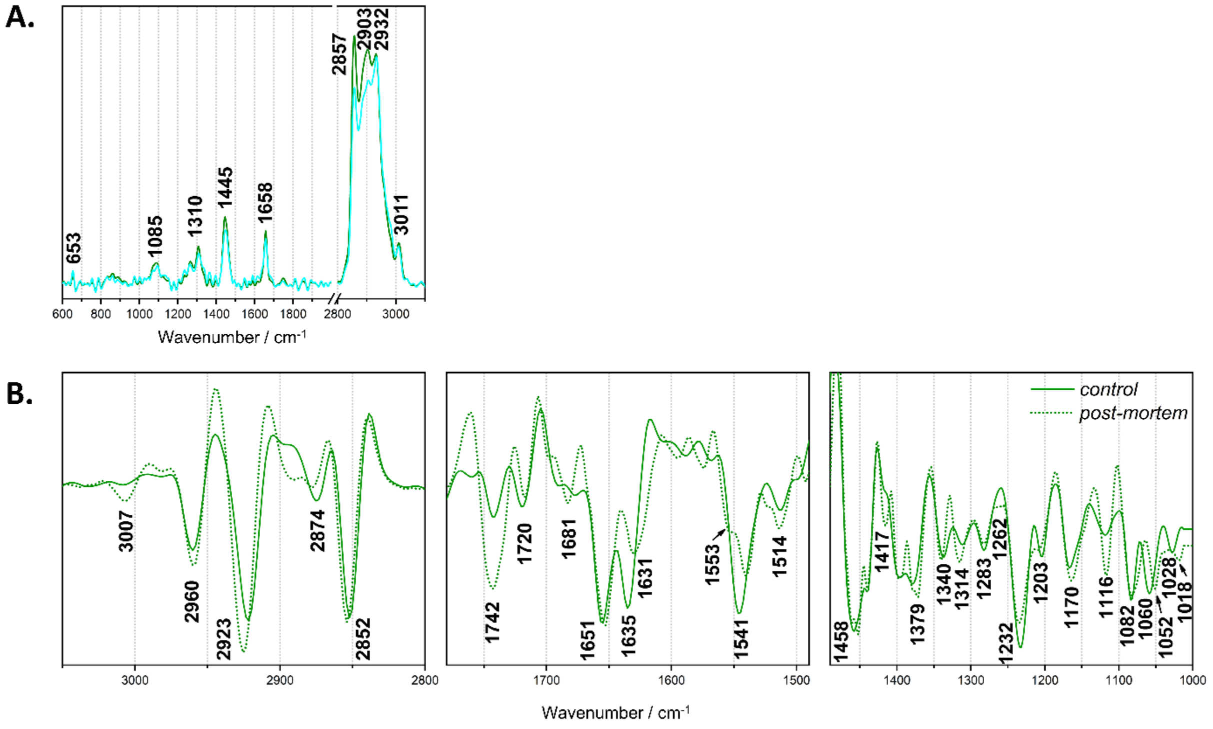

FIGURE S5. (A) Mean Raman spectra with band positions extracted from the KMCA analysis are given in Figure S3B. (B) Mean FTIR spectra extracted from the UHCA analysis of the columnar epithelium (green classes) from HC (solid trace, Fig. S2C) and PM samples (dashed trace, Fig. S3C).

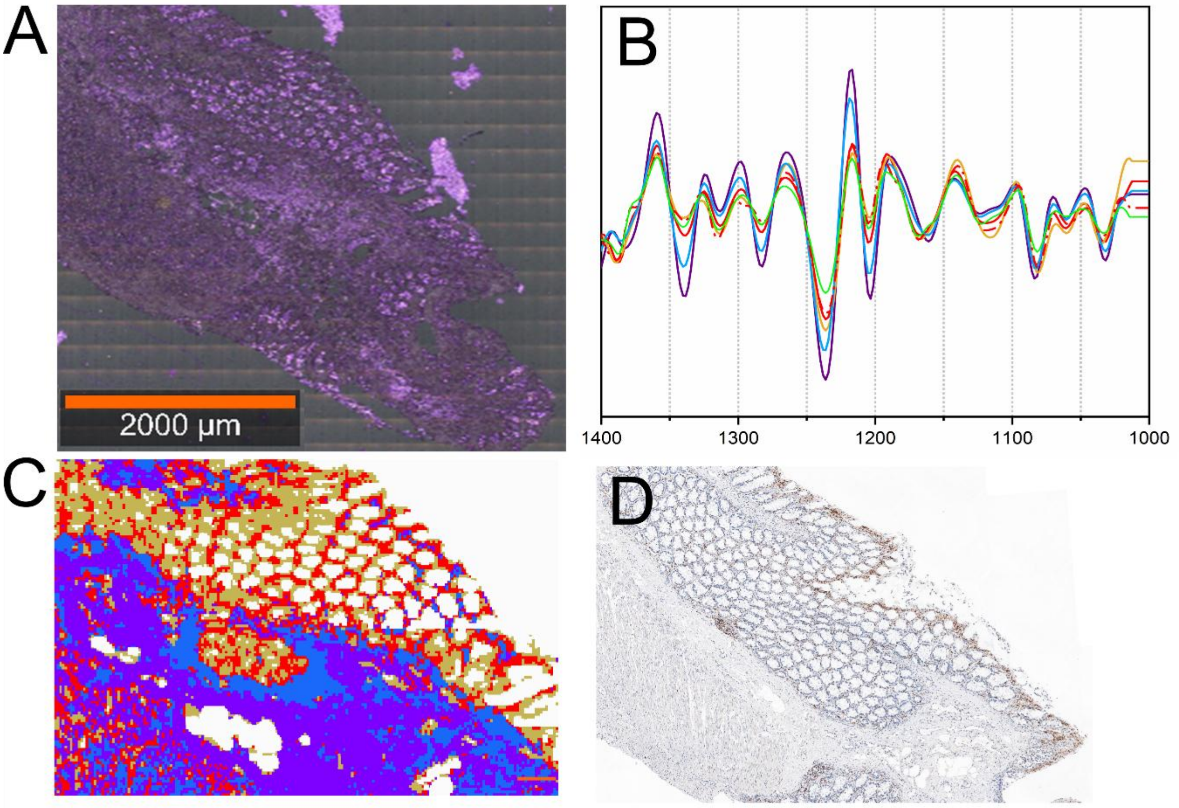

FIGURE S6. A white-light image of post-mortem FFPE intestinal cross-sections of the sigmoid colon showing region of interest (ROI) with a higher distribution of immune cells (A, 1400 µm × 2100 µm) selected for FTIR imaging (20× magnification); B, The mean second derivative IR spectra for ROI (a red dashed trace from ROI of low inflamed colonic mucosa; please see Figure 5); blue - submucosa and serosa, red - immune cells and lymphoid tissue, violet - smooth muscles, light green - lamina propria and connective tissue (down). C, Corresponding UHCA false-color cluster map. D, Matched, post-mortem colonic mucosa showing immunohistochemical staining against $CD3^+$ immune cells (5x magnification).

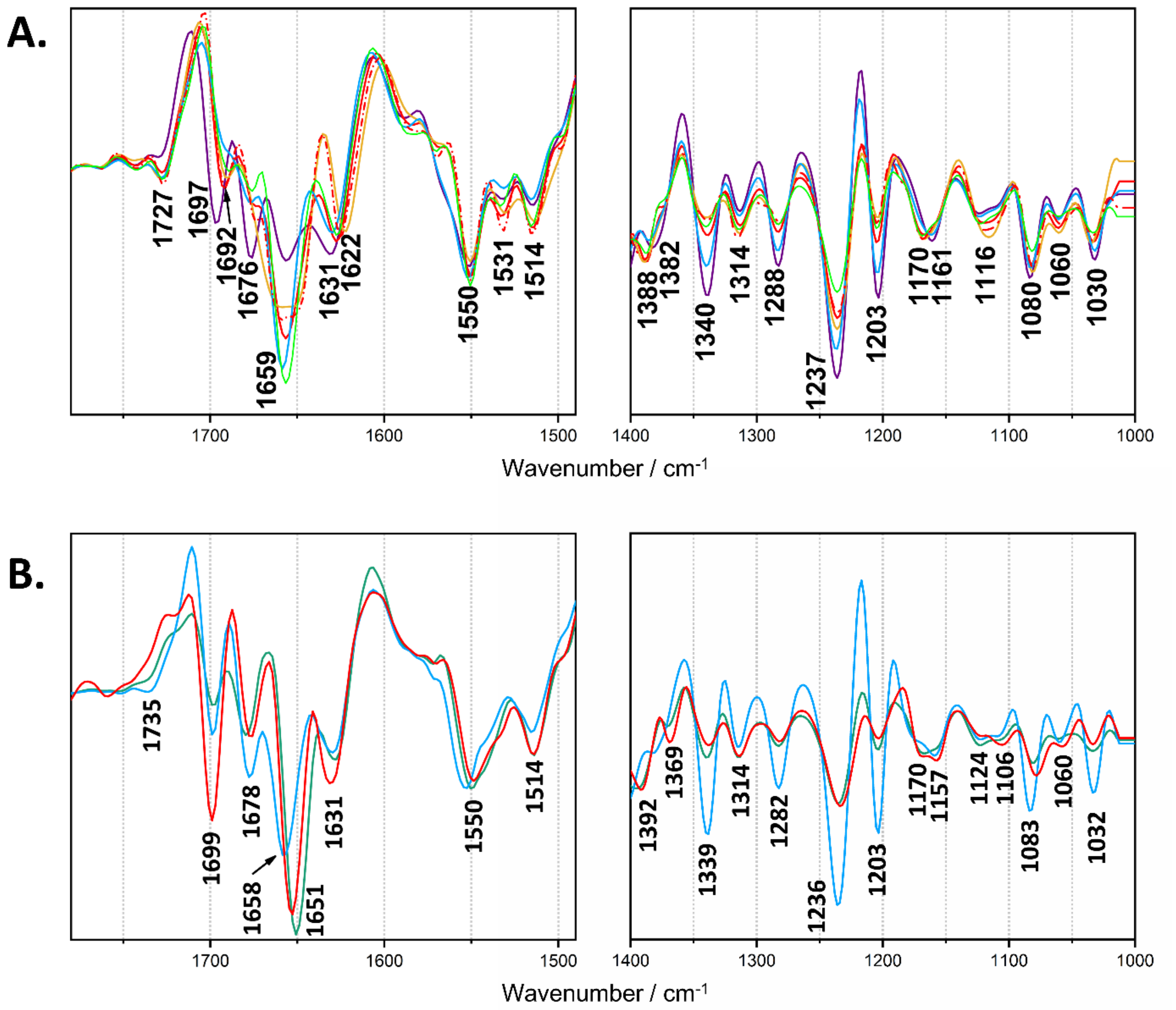

FIGURE S7. Mean FTIR spectra (A and B) with band positions extracted from the UHCA analysis are given in Figs. 6C and 5D, respectively. Dashed red and solid beige traces in (A) correspond to immune cells and lymphoid tissue differentiated by the UHCA analysis (Fig. 5 A2).

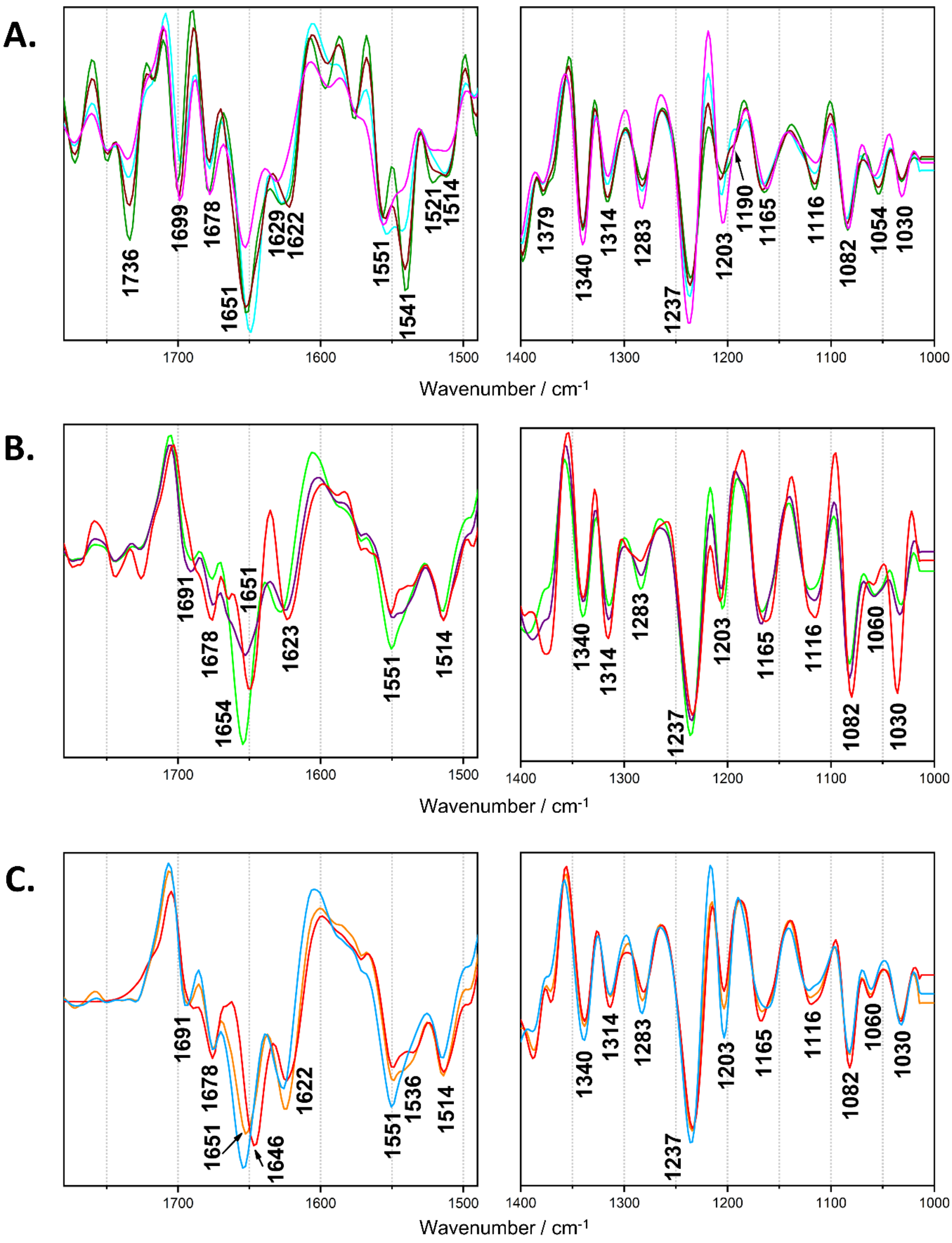

FIGURE S8. Mean FTIR spectra (A-C) with band positions extracted from the UHCA analysis are given in Figure 6A-C, respectively.
